# Evidence of spillovers from (non)cooperative human-bot to human-human interactions

**DOI:** 10.1016/j.isci.2025.113006

**Published:** 2025-06-25

**Authors:** Ashley Harrell, Margaret L. Traeger

**Affiliations:** 1 Department of Sociology, Duke University, Durham, NC, USA; 2 Department of Information Technology, Analytics, and Operations, University of Notre Dame, Notre Dame, IN, USA

**Keywords:** social sciences

## Abstract

It is well-documented that cooperation spills over among humans: people’s cooperative choices are influenced by their (non)cooperative alters, even in downstream interactions with new partners. We ask: do (non)cooperative interactions with bots spill over to subsequent interactions with humans, and if so, how? Across two pre-registered experiments (combined *N* = 83,411 decisions by 4,171 participants told they are interacting with a bot or human), we demonstrate that human-bot interactions do spill over to human-human ones, and in two ways. First, interacting with a bot reduces cooperation not only during the initial interaction, but also reduces downstream cooperation toward a new human partner. Additionally, bots’ (like human partners’) behavior matters: interactions with bots playing tit-for-tat promote cooperation, and interactions with noncooperative bots reduce cooperation toward downstream human partners. The implementation of bots in previously human-only spaces alters human cooperation, not just toward bots, but toward other humans as well.

## Highlights

• Human-bot interactions spill over to downstream human-human ones
• Cooperative bots promote cooperation, while noncooperative bots reduce it
• Interactions with bots lower empathic concern, perspective-taking, and cooperation

## Graphical abstract

**Figure:**
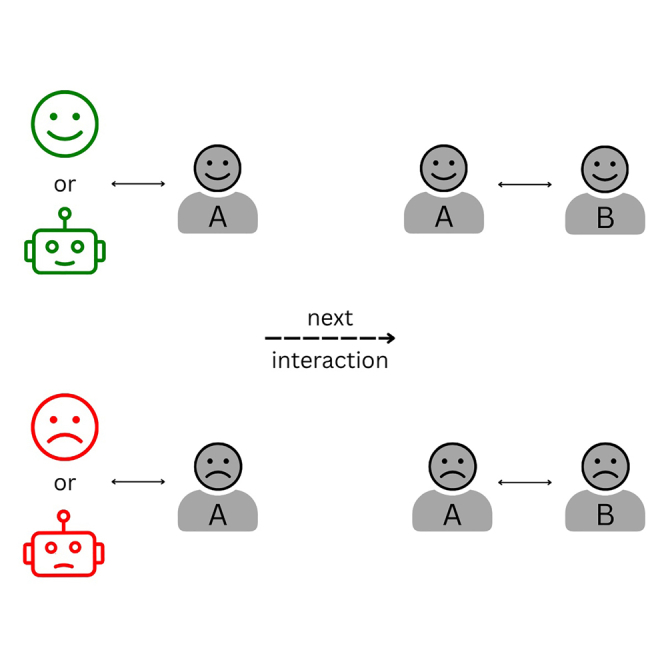

## Introduction

Cooperation is essential to functional societies. From team projects in the workplace to following local traffic laws to managing the spread of a global pandemic, people often must forgo their short-term self-interest for the good of the collective. Prior work has demonstrated that cooperation “spills over” from person to person: the influence of a partner’s cooperative or selfish behavior ripples through to one’s subsequent treatment of new alters.^1^^,^^2^^,^^3^^,^^4^^,^^5^^,^^6^ This and related work demonstrates the powerful role that connections play in fostering cooperation.^2^^,^^7^^,^^8^^,^^9^^,^^10^^,^^11^^,^^12^^,^^13^

While there is clear evidence that cooperative behavior spills over from human to human, an array of technologies is encroaching into more and more human spaces.^14^^,^^15^^,^^16^ As a result, we are now– and will be in the foreseeable future– engaging in collaborative decision-making with (ro)bots, computer programs, and forms of AI at work,^17^ on the road,^18^^,^^19^ and in our efforts to combat pandemics^20^ in “hybrid systems.”^19^^,^^21^ Despite exciting recent developments in the study of human behavior within hybrid systems, relatively little is currently known about how interacting with these technologies shapes subsequent human-human interactions.^22^^,^^23^ In the studies described here, we integrate prior work demonstrating that spillover effects occur from *person* to person with developments in the field of human-AI interaction. We test whether (non)cooperative interactions with *bots* spill over to subsequent cooperative interactions with other people – and if they do so in the same way that cooperative spillovers occur from *person* to person.

Because we are increasingly tasked with interacting in hybrid systems, recent work has sought to understand how human-machine interactions impact social behavior during the interaction itself.^24^^,^^25^ Much of this research suggests that humans’ behaviors in their interactions with technology– from simple bots to more complex AI– are remarkably similar to their behaviors in human-human interactions, because they elicit similar assumptions for social roles and behaviors.^23^^,^^26^^,^^27^^,^^28^^,^^29^ People anthropomorphize and build relationships with AI.^30^ They respond positively to vulnerability displays made by robots in group conversation,^31^ and reciprocate cooperation extended by bots in a repeated Prisoner’s Dilemma.^32^ They also conform to decisions made by robots,^33^ trust robots,^34^ and retaliate against robots that violate their trust,^35^ at similar levels as they do other humans.

We hypothesize that, like interactions with humans, interactions with machines will spill over to affect one’s downstream treatment of human partners. That is, in line with prior research on behavioral spillovers,^1^^,^^2^^,^^3^^,^^4^^,^^5^^,^^6^ if people respond to *bot* behavior in similar ways as they do *human* behavior, we expect that (non)cooperative interactions with a bot will foster downstream (non)cooperation, even toward new human partners. Understanding whether behavioral spillovers occur from bot to human as they do from human to human, and what type of behaviors (cooperative, noncooperative, or both) spill over, is critical. On the one hand, it might suggest that interacting with machines that are programmed to follow cooperative norms will have beneficial effects on downstream human-human cooperation, in the same way that interacting with a cooperative human partner does. Likewise, if selfishness spills over from bot to human, it suggests that interacting with noncooperative AI may harm subsequent human-human interactions. In this way, as in human-only social interactions, norms about cooperation may be transmitted through interactions with non-human entities as well.

Another stream of research suggests an additional (non-mutually exclusive) path by which interactions with bots might affect downstream interactions with human partners: that directly interacting with a bot (compared to a person) will lead to a lasting reduction in cooperation toward human partners, regardless of the bot’s (cooperative or noncooperative) strategy. Prior work examining how humans interact with technology in cooperative tasks such as the Prisoner’s Dilemma finds that people are generally less cooperative toward bots^32^^,^^36^^,^^37^^,^^38^^,^^39^^,^^40^ and feel less guilt about exploiting machines than they do other people.^41^ This is because prosocial actions such as cooperation are fostered by empathic concern and perspective-taking,^42^^,^^43^^,^^44^ social-emotional competencies toward other *humans* that help people navigate mixed-motive settings.^45^ Empathic concern and perspective-taking are emotional and cognitive subsets, respectively, of *empathy*, which vary across people as a trait.^46^ But they can also be state-based, measured toward a given alter at a given time.^42^^,^^47^^,^^48^ Here we argue that people will have reduced empathy (emotional, cognitive, or both) and, in turn, cooperation when they interact with a bot, compared to when they interact with a person. And if people do not compartmentalize their interactions with bots from their interactions with humans (which we argue is unlikely, given the work described above), this reduction in empathy and (in turn) cooperation while interacting with bots might continue to suppress people’s downstream cooperation with other humans, regardless of the bot’s strategy.

While research on spillovers from human-bot to human-human interactions is relatively limited, a recent set of experiments demonstrates that interacting with an unfair algorithm in a Dictator Game reduces people’s willingness to engage in prosocial punishment toward an unfair human in a subsequent task.^22^ We note that the finding that an antisocial AI can lead to downstream antisocial behavior is consistent with both pathways proposed above. Antisocial algorithmic behavior leading to antisocial human behavior is consistent with behavioral spillover. It is also possible that interacting with an AI *in general* – even if it had behaved neutrally or prosocially– could *also* have caused downstream antisocial behavior, in line with the arguments above about reductions in empathy after interacting with a bot. Because this work sought to test different questions, participants did not interact with a neutral or prosocial AI as a comparison. To expand on this work, our experiments assess how dyadic (non)cooperative interactions with a bot (versus a human) impact downstream (non)cooperative dyadic interactions with a new human partner. This allows us to explore the two distinct spillover pathways we have proposed above.

In sum, we test how a primary cooperative or noncooperative interaction with a bot (versus with another human) influences the treatment of another human in a downstream interaction– and, if so, whether it occurs via what we refer to as “behavioral spillover” (i.e., (non)cooperative interactions promote (non)cooperative behaviors in subsequent interactions with humans), “emotional spillover” (i.e., interactions with a bot reduce cooperation in subsequent interactions with humans), or both. In Study 2, we more directly assess whether interacting with a bot reduces cooperation with humans downstream by suppressing empathy.

**Figure 1:**
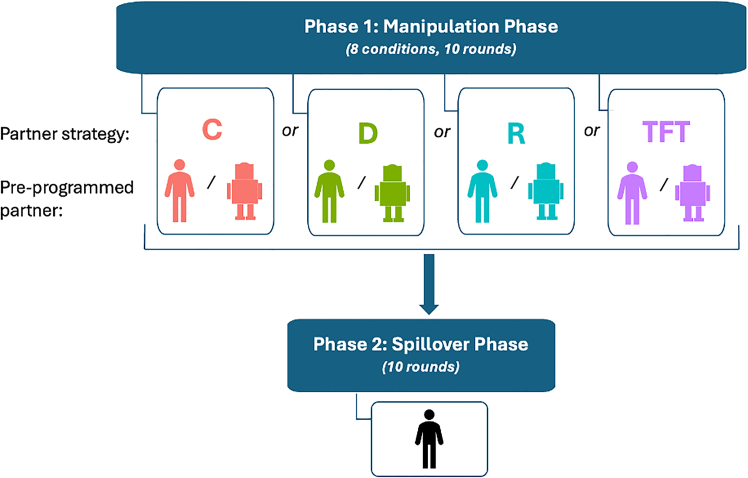
Outline of the basic design for both studies C, always cooperate; D, always defect; R, random; TFT, tit for tat.

We conducted two pre-registered experiments (combined *N* = 83,411 cooperation decisions made by 4,171 unique participants); the basic study design is shown in Figure 1. In both studies, U.S. adult participants first completed 10 rounds of a repeated Prisoner’s Dilemma Game (PDG) with an alter they were told in advance was either a bot or a human, randomly assigned. In reality, both “types” of partners were pre-programmed; we call this set of interactions the *manipulation phase*. The deception^49^^,^^50^^,^^51^ allowed us to hold constant the manipulation phase alter’s behavior across conditions, and therefore assess the effect of the first alter’s “type” (i.e., human or bot) on participants’ subsequent behavior with a new human partner, rather than the effect of any difference in decisions made by a computer program versus a real other participant’s behavior. After they completed the entire study, participants were fully debriefed about the minor deception for some participants during the first phase of the study, in line with best-practice recommendations and prior work showing that being deceived in one study does not affect the validity of others.^52^^,^^53^^,^^54^

In the manipulation phase, the pre-programmed alter was randomly assigned to use one of four strategies: *always cooperate* (C in Figure 1), *always defect* (D), *random* (R), or *tit for tat* (TFT). This tightly controlled, simple bot behavior allowed us to clearly observe how each of our conditions affected our behavioral outcomes of interest. In both studies, we evaluated how (1) interacting with a bot versus an ostensible human alter, and (2) the alter’s strategy, shaped our focal participants’ decisions with a human (a new, actual other participant) in a downstream interaction, which we call the *spillover phase* (see Figure 1). In the spillover phase of Study 1, participants were always paired with someone who had been in their same condition in the manipulation phase (e.g., both had previously been paired with a bot that always cooperated). In the spillover phase of Study 2, we paired participants without regard to their manipulation phase condition, as a more conservative test of spillover effects (see the next section for more details).

Study 2 also included additional items measuring empathy toward the partner. We adapted the empathic concern and perspective-taking subscales from prior work^46^ as measures of the emotional and cognitive aspects of empathy, respectively. For empathic concern, we adapted a scale from other prior experiments assessing empathy toward one’s partner after a manipulation^42^^,^^47^^,^^48^ (sample item from our study: feeling “compassionate” after finding out that they would be paired with “Participant M” or “Robot M”). The perspective-taking scale was also adapted from prior work assessing perspective-taking toward a specific partner^42^; it asked participants to what extent they were, e.g., “making an effort to see the world through [the partner’s] eyes” after learning that they would be paired with [the partner] (full scales available in the supplemental information). To assess the proposed mediation process, we measured these items after participants found out who their partner was (i.e., a bot or human), but before they began to make decisions with the partner in the PDG.

## Results

**Figure 2:**
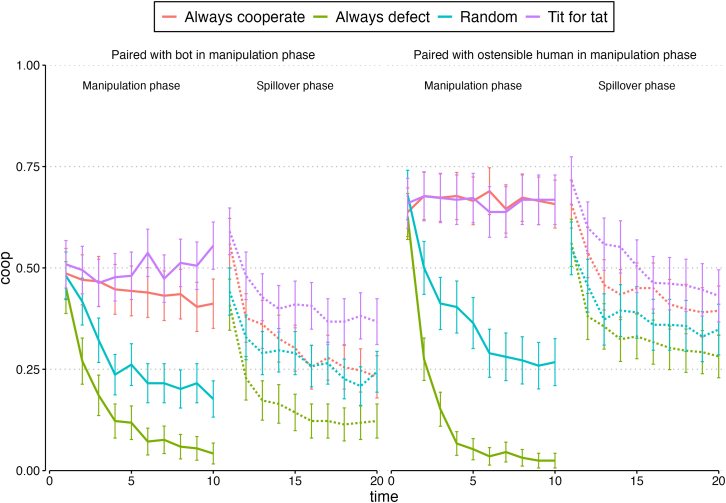
Proportion cooperating by condition, Study 1 Error bars represent 95% confidence intervals.

Over the two phases of Study 1, we observed 41,053 cooperation decisions made by 2,053 participants, 44.3% of whom were female (our preregistered target sample size was 2,000). Figure 1, Figure 2, Figure 3 in the supplemental information provides information about our sample demographics for both studies. We used logistic generalized linear multilevel models with random intercepts to account for the dependencies in the nested data (multiple rounds were nested in participants, who were nested in dyads in the spillover phase). Because including them improved model fit and because our key models include cross-level interactions (i.e., round by condition), our models also include random slopes at the lower (round) level. The outcome variable was cooperation (vs. defection).

We followed our pre-registered analysis plan by simultaneously assessing the main effects of our two key manipulations (i.e., the manipulation phase partner’s *strategy* and whether or not the manipulation phase partner was a *bot*), conducting follow-up models with interactions between conditions, interactions between our conditions and round (i.e., time), and three-way interactions between our conditions and round, and retaining the model that yielded the best fit according to the AIC and chi-squared goodness-of-fit tests. We also conducted bootstrapping analyses on our models (*N* = 1,000 simulations, percentile method), and provide the bootstrapped 95% confidence intervals around our reported odds ratios in brackets. As another robustness check, because our study included analyses across two separate phases in each of two studies, we assessed adjusted *p*-values using the False Discovery Rate (FDR) adjustment^55^^,^^56^^,^^57^ on our key models. The implementation of FDR changed the significance of only one result we report; it is noted below and does not alter our key findings.

Figure 2 shows descriptive cooperation patterns across the 20 rounds of Study 1. Alternatively, Figure S1 in the supplemental information shows broader within-participant cooperation patterns (i.e., not just whether they cooperated or defected in any given round, but whether they, for instance, played Tit for Tat with their partner) across the rounds of the manipulation and spillover phases.

### Study 1, manipulation phase

Beginning with the *manipulation phase*, our best-fitting model included terms for condition, round, and the two- and three-way interactions (see the supplemental information, Table S2, Model 1). First, we find that the pre-programmed partner had the expected behavioral effects: holding constant whether the alter was a bot or ostensible human, participants were more likely to cooperate when their pre-programmed partner played Tit for Tat, both overall (OR = 2.53 [1.40, 4.54], *p* < 0.01) and increasingly over time (OR = 2.03 [1.85, 2.49], *p* < 0.001), compared to when their partner made choices at random (see also Figure 2, where cooperation dropped over time when the partner made choices at random, but stayed high across the manipulation phase rounds when the partner played Tit for Tat). Participants were also more likely to cooperate when their partner always cooperated, both overall (OR = 2.34 [1.33, 4.29], *p* < 0.01) and increasingly over time (OR = 2.01 [1.82, 2.42], *p* < 0.001). They were also *less* likely to cooperate with their alter when the alter always defected, both overall (OR = 0.56 [0.34, 0.99], *p* < 0.05) and over time (OR = 0.46 [0.38, 0.53], *p* < 0.001), compared to when the pre-programmed partner made choices at random.

Now consider differences based on whether the partner was a bot or an ostensible human. In line with prior work,^32^^,^^36^^,^^37^^,^^38^^,^^40^ and holding constant the alter’s strategy, participants were less cooperative when they were told their alter was a bot (OR = 0.35 [0.21, 0.60], *p* < 0.001). We also observed two significant three-way interactions, suggesting that cooperation patterns with bots (versus ostensible humans) changed across the rounds of the study, depending also on the partner’s strategy. First, over time, participants were less likely to cooperate with bots that always cooperated, compared to ostensible humans who always cooperated (OR = 0.69 [0.56, 0.81], *p* < 0.001). While a follow-up simple slopes analysis revealed that partners who always cooperated maintained higher cooperation than partners who behaved at random during all rounds of the phase, the drop in cooperation for those paired with an always-cooperating bot (compared to those paired with an always-cooperating ostensible human) can be seen in Figure 2. This finding is in line with prior work suggesting that humans are willing to exploit cooperative bots more than they are to exploit cooperative people.^36^

Second, over time, participants became *more* likely to cooperate with bots who always defected, compared to ostensible humans who always defected [OR = 1.62 [1.32, 2.04], *p* < 0.001). Simple slopes analyses revealed that by round 3, cooperation with an always-defecting partner was similar regardless of whether the partner was a bot or an ostensible human; by round 7, cooperation with an always-defecting partner was higher when the partner was a bot. This finding was less expected; it may stem from participants' attempts to “game” the bot into reciprocating cooperation, or accepting noncooperative behavior from bots in a way they do not from humans.

As a robustness check on our key models, we also conducted exploratory (i.e., not pre-registered) Cox proportional hazards models to examine whether time to first defection differed by condition. The results aligned with those reported above: participants whose manipulation phase partner always defected themselves defected significantly faster (HR = 1.35 [1.23, 1.48], *p* < 0.001), while those whose partner played Tit for Tat (HR = 0.47 [0.41, 0.55], *p* < 0.001) or always cooperated (HR = 0.49 [0.42, 0.56], *p* < 0.001) were significantly slower to defect, compared to those whose partner made choices at random (see Model 1 in Table S3). Additionally, holding the partner’s strategy constant, participants paired with a bot defected sooner than those paired with an ostensible human partner (HR = 1.52 [1.39, 1.66], *p* < 0.001).

Model 2 in Table S2 shows that controlling for participant age and gender did not impact our key results. Likewise, Table S4 shows that neither gender nor age interacted with being paired with a bot to differentially impact cooperation. We conducted these exploratory analyses because it is plausible that both age and gender impact feelings toward AI, including the bots used in our study, to *differentially* impact cooperation by condition (i.e., such that age or gender interacted with being paired with a bot versus ostensible human to predict cooperation). For instance, some prior work has shown that women are more trusting of an autonomous security robot than men.^58^ Other work, however, has suggested the opposite: that women are *less* trusting of robots than men.^59^^,^^60^ These findings, though conflicting, suggested that we also ought to assess whether the effect of being partnered with a bot– on either cooperation with the bot (in the manipulation phase) or cooperation with a downstream partner after being partnered with a bot (in the spillover phase)– was altered by participant gender. Likewise, prior work suggests that age is associated with more negative attitudes toward AI.^61^ Thus, the effect of being in the *bot* condition on cooperation might also have differed by participant age. Instead, our main findings held; this suggests that both men and women were less likely to cooperate with bots than ostensible people, and that this was the case regardless of participant age.

### Study 1, spillover phase

Our primary research questions are centered on what happened in the spillover phase: whether being partnered with cooperative and noncooperative bots (versus cooperative and noncooperative humans) spills over to a *subsequent* partnership (i.e., whether the two manipulations we introduced in the manipulation phase had lasting effects in the spillover phase; see the dashed lines in Figure 2) with a new human partner.

Again, we assessed models with just main effects, interactions between our conditions, and interactions between our conditions and time (along with three-way interactions). These models also controlled for the focal participant’s behavior in the previous (manipulation) phase. Controlling for the focal participant’s own manipulation phase behavior allows us to assess whether spillover effects occurred above and beyond the ego’s baseline behavior.

The best-fitting model of cooperative choices (see the supplemental information, Table S5, Model 1) included terms for our conditions, round, and the condition *x* round interactions (but not interactions between our conditions or three-way interactions between our conditions and round, none of which were significant). First, we find evidence for emotional spillover: the reduction in cooperation that we observed among those participants paired with a bot in the manipulation phase *persisted* into the spillover phase, even after controlling for the participants’ own manipulation phase behavior. Participants who had previously been paired with a bot cooperated less with their new, human spillover phase partner than those who had previously been paired with an ostensible human, both overall (OR = 0.48 [0.37, 0.64], *p* < 0.001) and over time (OR = 0.82 [0.70, 0.90], *p* < 0.01).

We also find evidence for behavioral spillover: the manipulation phase partner’s behavior affected our focal participants’ behaviors in the spillover phase. Holding constant whether the manipulation phase partner was a human or bot, as well as the focal participant’s behavior in the manipulation phase, those whose manipulation phase partner had been pre-programmed to always defect started out cooperating at similar rates as those whose previous partner had made choices at random. But across the rounds of the spillover phase, they dropped their cooperation, compared to those whose previous partner had played at random (OR = 0.66 [0.55, 0.80], *p* < 0.001). Follow-up simple slopes analyses revealed that the negative effects of having a previous partner who always defected occurred beginning in round 3 and persisted over all subsequent rounds.

Additionally, those whose previous partner played Tit for Tat continued to cooperate more in the spillover phase, beginning in the first round (main effect, OR = 3.11 [2.09, 4.56], *p* < 0.001) and increasingly over time across the rounds of the spillover phase (TFT x round interaction, OR = 1.20 [1.03, 1.48], *p* < 0.05; the interaction effect, but not the main effect, becomes non-significant at *p* = 0.05 when we adjust *p*-values using the FDR). Likewise, those whose manipulation phase partner always cooperated also cooperated more in the spillover phase (OR = 1.86 [1.24, 2.66], *p* < 0.01; the always-cooperate x round interaction was not significant), compared to those whose manipulation phase partner made choices at random.

As we noted briefly above, none of the interaction effects between the bot condition and the alter’s strategy conditions were significant in the spillover phase; nor did we observe any significant three-way interactions between our two key manipulations and round in the phase. This suggests that human-bot pairings in our study suppressed cooperation in downstream human-human pairs, and partner strategy affected downstream cooperation at similar magnitudes from the bot partner to the human as from the human partner to the human. Consistent with the manipulation phase results, Model 2 in Table S5 shows that controlling for participant age and gender did not impact our key results in the spillover phase. Likewise, Table S6 shows that neither gender nor age interacted with being paired with a bot to differentially impact cooperation in the spillover phase. Instead, our main findings held.

We also controlled for participants' answers to items measuring suspicion of the manipulation phase partner and the spillover phase partner in this model (see the next section for more details). None of our key findings were altered by controlling for suspicion of either partner (VIF for the two suspicion items was acceptable, both ≤1.40). We did not include suspicion controls in the models for the manipulation phase because suspicion of the manipulation phase partner was highly correlated with our key manipulation, i.e., whether the participant had been told their partner was a bot or a human.

Follow-up Cox proportional hazards models demonstrated that those whose previous (manipulation phase) partner had played Tit for Tat *remained* less likely to defect until a later round in the spillover phase (HR = 0.58 [0.49, 0.69], *p* < 0.001), as did those whose manipulation phase partner had always cooperated (HR = 0.75 [0.62, 0.85], *p* < 0.05), compared to those in the random condition. Those who had partners in the manipulation phase who always defected were no more likely to defect compared to those who had a partner that played at random (HR = 1.09 [0.92, 1.22], *p* = 0.50). Participants who had previously been paired with a bot continued to defect in earlier rounds in the spillover phase, compared to those who had previously been paired with an ostensible human (HR = 1.55 [1.37, 1.72], *p* < 0.001; see Model 2 in Table S3).

### Study 1, suspicion

Finally, at the end of the study (i.e., only once both phases were complete), we asked participants two follow-up questions: to what extent the participant thought their *first* partner (the *manipulation phase partner*, who they had been told was either a bot or human participant, depending on condition) and their *second* partner (the *spillover phase partner*, who they had been told, correctly, was another participant) was “a real person,” on a one to seven scale. Our goal was to assess a possible competing explanation for why interacting with a bot reduced cooperation with humans in a subsequent phase. Specifically, participants who had been told they were interacting with a bot in the first phase may have been more suspicious that their second phase partner was an actual other human participant (even though the partner *was* a real other participant), compared to participants who had been told they were interacting with a person in the first phase. As a result, those told they were interacting with a bot may have cooperated less with their subsequent human partner at least partly because they did not believe the subsequent human partner was “real.”

Of course, we should expect differences by condition in participant responses about the first partner. For the manipulation phase partner, this question was like a manipulation check: those in the bot condition would be expected to have significantly lower scores on this item than those in the human condition. However, if their response about the *second* partner *also* differed by condition, we would not be able to rule out whether differences in cooperation were driven, even partly, by differences in suspicion that the spillover phase partner was another participant. As expected, holding constant our other condition (the partner’s strategy), participants in the *bot* condition were significantly less likely than those in the *human* condition to say their manipulation phase partner was a human (Model 1 in Table S7, *B* = −2.38 [−2.54, −2.23], *p* < 0.001). This suggests that our manipulation was effective: those in the *human* condition believed their manipulation phase partner was more of a real person than those in the *bot* condition.

We also tested for interactions between our conditions and suspicion. Participants reported less suspicion of their manipulation phase partner (MPP) when they were an ostensible human who played Tit for Tat (main effect of *MPP played Tit for Tat*, *B* = 0.47 [0.07, 0.88], *p* < 0.01, see Model 1 in Table S8), but not when they were a known bot who played Tit for Tat (interaction effect of *bot x MPP played Tit for Tat*, *B* = −0.49 [-0.95, −0.04], *p* < 0.05. A goodness-of-fit test suggested that included the interactions improves model fit (Χ^2^ (3) = 42.53, *p* = 0.003). We interpret this as more support for our manipulation check: while the ostensible human’s strategy influenced participants’ suspicion of them, the partner’s strategy did not matter when participants were explicitly told that they were playing with a programmed bot. None of the other *MPP was a bot x MPP strategy* interactions significantly predicted suspicion of the manipulation phase partner.

However, participants who had previously interacted with a bot (i.e., those in the *bot* condition) were also more suspicious of their spillover phase *human* partner, compared to those in the *human* condition (Model 2 in Table S7, *B* = −0.29 [−0.47, −0.10], *p* < 0.01). None of the condition interactions significantly predicted suspicion of the spillover phase partner (Model 2 in Table S8). Thus, in Study 1, we could not rule out that participants who had previously interacted with a bot were less cooperative with their human partner because they were suspicious that the human partner was not real.

We made changes to Study 2 to address this issue. Specifically, Study 1 provides initial evidence for our arguments. But note that our Study 1 participants were paired with a spillover phase partner who had been in their same condition in the manipulation phase. Thus, our behavioral spillover effects may have been reinforced by the new partner having previously been in the same condition and having experienced the same behaviors from the previous partner. We conducted a more conservative test of spillover effects in Study 2: participants were paired with a spillover phase partner at random, rather than a spillover phase partner who had been in their same condition.

Just as pairing participants with someone in their same condition might have magnified spillover effects, it could also have magnified the differences in suspicion by condition that we found in Study 1. For example, two participants paired with each other who had both been previously paired with an always-defecting bot in the manipulation phase might have started the spillover phase so uncooperatively, it would be easy for them to conclude that they were simply paired with another always-defecting bot, even though they were paired with a real person (each other). We suspected that by pairing participants without regard to condition, their experience in the spillover phase would be more natural and less suspicious.

Finally, we also conducted Study 2 to further probe what we have labeled “emotional spillover”: that being partnered with a bot in the manipulation phase had lasting negative effects on cooperation with a human in the spillover phase. Specifically, in Study 2, we directly assessed participants’ *empathic concern* and *perspective-taking* (i.e., emotional and cognitive components of empathy, respectively) toward their partner by adapting scales from prior work. These scales asked participants about their empathic concern (sample item: feeling “compassionate” after finding out they “will be paired with Participant W”) and perspective-taking (sample item: “I am making an effort to see the world through Robot M’s eyes”) *after* they found out that the partner was a bot or human, but before the PDG began. We aimed to test whether the relationship between previously being paired with a bot and reduced cooperation in the spillover phase was mediated by a reduction in empathic concern and perspective-taking among participants who had previously been paired with a bot.

### Study 2, manipulation phase

**Figure 3:**
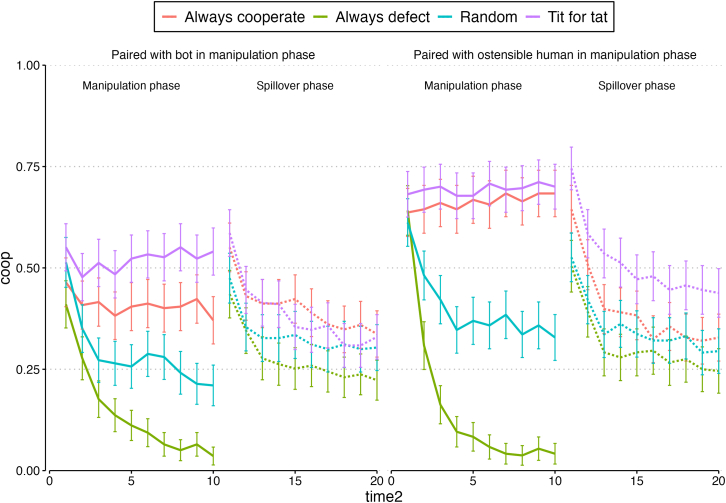
Proportion cooperating by condition, Study 2 Error bars represent 95% confidence intervals.

Over the two phases of Study 2, we observed 42,358 cooperation decisions made by 2,118 participants, 52.6% of whom were female (our preregistered target sample size was 2,000). Figure 3 shows cooperation decisions made across the study; the patterns look very similar to those in Study 1. Likewise, Figure S2 in the supplemental information shows broader within-participant cooperation patterns across the rounds of the manipulation and spillover phases.

Beginning with the manipulation phase, consistent with Study 1, the best-fitting model of participants' round-wise cooperative decisions included terms for condition, time, and the two- and three-way statistical interactions (see the supplemental information, Table S9, Model 1). Holding constant whether the partner in this phase was a bot or an ostensible human, we again find the expected effects of the pre-programmed partner’s strategy. Participants were more likely to cooperate in the manipulation phase when their pre-programmed manipulation phase partner played Tit for Tat, both overall (OR = 3.99 [2.33, 6.20], *p* < 0.001) and increasingly across the rounds of the study (OR = 1.60 [1.48, 1.85], *p* < 0.001), compared to when their partner made choices at random. They were also more likely to cooperate when their partner always cooperated, both overall (OR = 2.62 [1.58, 4.09], *p* < 0.001) and increasingly over time (OR = 1.60 [1.49, 1.82], *p* < 0.001). And, over time, they were less likely to cooperate when their pre-programmed partner always defected (OR = 0.50 [0.44, 0.55], *p* < 0.001; unlike Study 1, this pattern only emerged over time rather than as a main effect), compared to when the partner made choices at random. A follow-up simple slopes analysis reveals that this difference was present by the second round and continued over the remaining rounds of the spillover phase.

We also replicated our Study 1 finding that participants were less cooperative when their alter was a bot (holding constant the alter’s strategy), compared to when their alter was an ostensible human (OR = 0.52 [0.34, 0.83], *p* < 0.001). Unlike in Study 1; interactions between our conditions in the manipulation phase suggested that participants were especially less likely to cooperate with a known bot who played Tit for Tat (OR = 0.47 [0.26, 0.89], *p* < 0.05) or always cooperate (OR = 0.42 [0.23, 0.82], *p* < 0.05). These findings, however, suggest that participants were exploiting cooperative bots,^36^ which we did find evidence for in Study 1 via the three-way interaction. We also observed this same three-way interaction, suggesting that people were less likely to cooperate with bots who always cooperated across the rounds of the phase (OR = 0.77 [0.66, 0.87], *p* < 0.001), as in Study 1. Likewise, participants were again more likely to cooperate with bots that always defected over time, compared to ostensible humans that always defected (OR = 1.44 [1.24, 1.71], *p* < 0.001).

As with Study 1, we checked the robustness of the results with Cox proportional hazards models. The results aligned with those reported above: participants whose manipulation phase partner played Tit for Tat (HR = 0.45 [0.39, 0.52], *p* < 0.001) or always cooperate (HR = 0.54 [0.47, 0.62], *p* < 0.001) took longer to defect compared to those whose partner made choices at random, while those whose partner always defected (HR = 1.27 [1.16, 1.39], *p* < 0.001) defected sooner. Again, whether the partner was a bot or an ostensible person predicted time to defect, too: participants defected more quickly against a bot partner than an ostensible human one (HR = 1.57 [1.43, 1.73], *p* < 0.001; see Model 1, Table S10).

As in Study 1, Model 2 in Table S9 shows that controlling for participant age and gender did not impact our key results in Study 2. Likewise, Table S11 shows that neither gender nor age interacted with being paired with a bot to differentially impact cooperation.

### Study 2, spillover phase

Again, we turn to our key question: whether being paired with cooperative and noncooperative bots (versus cooperative and noncooperative humans) spills over to *subsequent* pairings with other people. The best fitting model of cooperative behavior in each round included terms for our conditions, time, and the condition *×* time interactions (but not interactions between our conditions or three-way interactions between our conditions and time). It is that model we report here (see the supplemental information, Table S12, Model 1). First, we again find evidence for emotional spillover. The negative effects of being partnered with a bot in the manipulation phase persisted into the spillover phase: controlling for their own baseline behavior in the manipulation phase, participants were, overall, less cooperative with a human participant when they had previously been partnered with a bot (OR = 0.74 [0.64, 0.89], *p* < 0.001).

Second, we assessed behavioral spillover by examining whether the manipulation phase partner’s behavior affected decisions made with a different partner in the spillover phase. As in Study 1, all else equal, those whose manipulation phase partner played Tit for Tat, or whose manipulation phase partner always cooperated, continued to cooperate more in the spillover phase (main effects of strategy, OR = 1.93 [1.51, 2.38], *p* < 0.001 and OR = 1.60 [1.26, 1.98], *p* < 0.001 respectively). But, likely reflecting the differences in the design of Study 1 and Study 2, the spillover effects of having a cooperative partner (i.e., either Tit for Tat or always cooperate) in the previous phase appeared to decrease over time, unlike in Study 1 (strategy × round interactions, OR = 0.89 [0.83, 0.97], *p* < 0.01 and OR = 0.89 [0.82, 0.97], *p* < 0.01 respectively). Follow-up analyses suggested that the spillover benefits of having a cooperative partner in the manipulation phase persisted into the initial rounds of second interaction, but dissipated halfway through the spillover phase (i.e., in round 5) for Tit for Tat and sooner for those who had a partner that always cooperated (round 3).

Finally, as in Study 1, those whose manipulation phase partner always defected began the interaction by cooperating at similar levels (OR = 1.05 [0.84, 1.33], *p* = 0.72) as those whose previous partner had made choices at random. But they dropped their cooperation over time in the spillover phase (OR = 0.88 [0.81, 0.96], *p* < 0.01); by round 4, they were cooperating at significantly lower rates than their counterparts whose manipulation phase partner had made choices at random. Thus, our results for behavioral spillover suggest that the benefits of having a cooperative partner do spill over, but fade over time– while having a noncooperative partner is associated with reduced cooperation that emerges and lasts over the rounds of interaction.

Again, none of the interactions between our bot condition and the strategy conditions were significant, nor were there any three-way interactions between the two manipulations and time. Thus, in Study 2 (just as in Study 1), both spillover processes occurred simultaneously and relatively independently. And again, follow-up Cox proportional hazards models checking the robustness of the results were highly consistent: as with Study 1, we again find that those whose manipulation phase partner had previously played Tit for Tat (HR = 0.61 [0.43, 0.66], *p* < 0.001) or had always cooperated (HR = 0.79 [0.63, 0.96], *p* < 0.05) remained slower to defect against their spillover phase partner. Being paired with a bot rather than an ostensible person in the manipulation phase also continued to lead to faster defection (HR = 1.39 [1.30, 1.77], *p* < 0.001; see Model 2 in Table S10). Across both studies, human-bot interactions suppressed cooperation in downstream human-human interactions, and partner strategy affected downstream cooperation at similar magnitudes from human to human and from bot to human. And again, controlling for age, gender, and suspicion of either partner did not impact our spillover phase results (Model 2 in Table S12); likewise, neither age nor gender interacted with being paired with a bot to differentially impact cooperation (Table S13).

### Study 2, suspicion

Suspicion patterns differed from Study 1, suggesting that the changes we made from Study 1 to Study 2 had the intended effect. Recall that in Study 1, participants who were paired with a bot were less likely to rate their partner as a real person, not only in the manipulation phase (as expected, since they were not paired with a real person in that phase), but also in the spillover phase (even though they were paired with a real person; this increased suspicion of the real human partner may explain their reduced cooperation toward them). In Study 2, again as expected, participants paired with a bot in the manipulation phase rated their manipulation phase partner as less of a real person than those whose manipulation phase partner was an ostensible human (Model 1 in Table S14, *B* = −2.03 [−2.18, −1.89], *p* < 0.001). But in the spillover phase, there were no differences in suspicion by condition (Model 2 in Table S14, *B* = 0.11 [−0.07, 0.28], *p* = 0.21).

As in Study 1, we also tested for interactions between our conditions and suspicion. For suspicion of the manipulation phase partner, none of the interactions were significant (Model 1 in Table S15). However, participants were especially more suspicious of their spillover phase partner (SPP) when they had previously been paired with a bot who had always defected (*B* = −0.52 [-1.03, −0.05], *p* < 0.05, Model 2 in Table S15). None of the other *SPP was a bot x SPP strategy* interactions significantly predicted suspicion of the spillover phase partner. This finding was not consistently observed across studies (i.e., we did not find it in Study 1), and a goodness-of-fit test suggested that including the condition interactions did not improve model fit (Χ^2^ (3) = 21.48, *p* = 0.17). Thus we retain the main effects only model in our main analyses.

### Study 2, mediation analyses

Finally, Study 2 allowed us to explore our proposed emotional spillover process more directly. Specifically, we argued that prosocial behaviors such as cooperation are fostered by empathy and perspective-taking, two distinct– and distinctly *human*– social-emotional competencies.^41^^,^^42^^,^^43^ Reduced cooperation with bots (compared to humans) may be driven by the concomitant reduction in empathy and perspective-taking that people feel toward bot partners, compared to human partners. In turn, this reduction in empathy and perspective-taking toward an *initial* bot partner (and subsequent reduced cooperation behavior with the bot partner, as we observed in both studies) may spill over to affect the treatment of subsequent *human* partners. We measured empathic concern and perspective taking after participants found out that their manipulation phase partner was a bot or human, but before the PDG began, in order to test these arguments.

The empathic concern and perspective-taking sub-scales were highly correlated (*r* = 0.53, *p* < 0.001), consistent with prior work.^46^^,^^62^ Thus, also consistent with prior work,^62^ we combined them by averaging the seven items into a single scale for *total empathy toward the manipulation phase partner*, which yielded high reliability (α = 0.87). Recall that being partnered with a bot in the manipulation phase predicted reduced cooperation rates with the spillover phase human partner, holding constant both the manipulation phase partner’s strategy and the ego’s own baseline behavior in the manipulation phase. We tested whether the anticipation of being partnered with a bot in the task was associated with reduced scores on the total empathy toward the partner scale, which, in turn, mediated the effects of previously being partnered with a bot on cooperation with the human partner in the spillover phase.

First, a linear regression revealed that participants who knew they would be paired with a bot in the manipulation phase reported less total empathy toward their (bot) partner (Table S16, Model 1, B = −0.91 [−1.02, −0.80], *p* < 0.001). In turn, empathy toward the manipulation phase partner predicted downstream cooperation with the *spillover phase* partner when we added empathy to our key model for the spillover phase of Study 2 (Table S16, Model 3, OR = 1.12 [1.06, 1.19], *p* < 0.001). After controlling for empathy toward the manipulation phase partner, the relationship between being partnered with a bot in the manipulation phase and cooperation with a human partner in the spillover phase was reduced, suggesting that empathy partially mediated the negative spillover effects (Model 3, OR = 0.82 [0.72, 1.00], *p* < 0.05). Goodness-of-fit tests suggest that the model with one term for total empathy toward the manipulation phase partner fits the data better than a model with only empathic concern, only perspective-taking, or both empathic concern and perspective-taking as subscales. In sum, knowing that one would be partnered in the PDG with a bot was associated with both reduced empathy toward the bot partner, which partially explained the lasting downstream effects when paired with a new, human partner.

## Discussion

Society is increasingly interwoven with bots, computer algorithms, and other forms of AI, which can shape human interactions.^21^^,^^24^^,^^63^ Our studies’ key message is that we must consider not only how human behavior is affected *during* human-machine interactions, but also how the initial human-machine interaction impacts *subsequent* interactions with other humans. In two large, pre-registered experiments, we evaluated how initial (non)cooperative interactions with a bot affected downstream human-human cooperation. Both studies revealed that the cooperative and noncooperative behaviors of both human *and* bot interactants spill over, influencing focal participants’ downstream treatment of other humans.

These findings support related work in the nascent field of how interactions with bots impact subsequent interactions with people.^22^^,^^23^^,^^31^ Our findings also lend further support to prior work showing that people are less cooperative toward bots than humans.^32^^,^^36^^,^^37^^,^^38^^,^^39^^,^^40^ We also add to and, critically, expand on this work by showing that the negative impacts of interacting with a bot persist into later cooperative interactions with other people. We found evidence for a lasting reduction in empathic concern and perspective-taking when interacting with bots, which spills over to new interactions with other humans.

Our results indicate that interacting with even simple bots can hinder the development of cooperation between people. Like human influences spread through networks,^64^ we find that the influence of bots also diffuses through social connections. These consequences could be particularly harmful if bots violate social norms, including those that humans have evolved for cooperation.^65^ At the same time, our results on behavioral spillovers suggest that interactions with relatively *cooperative* bots (i.e., those that follow conventional norms of cooperation, such as reciprocal tit for tat) may at least partly ameliorate these harms, promoting initial acts of cooperation in downstream human-human interactions. But the same goes for interactions with cooperative humans– they promote cooperation downstream, and without the negative emotional spillover effects we observed from interacting with bots. Future work might consider more complex hybrid networks– for instance, those where people can have multiple partners simultaneously, or can change their network ties– to determine whether the benefits of having a cooperative partner (whether bot or human) and the benefits of interacting with humans (over bots) may serve as complements to each other, promoting cooperation in larger hybrid networks.

Our findings imply that the design of AI systems is critical, as it can reinforce cooperative and non-cooperative behaviors in humans. Further, the results show how important it is that we question when the use of bots is ethically justified. If bots are capable of introducing more negative sentiment in human social systems, as we have shown here, accounting for the societal ramifications is paramount. The downstream effects of human-bot interactions on human-human ones– and managing those downstream effects– will become more important as humans engage more with bots. After all, AI is increasingly ubiquitous: writing news articles, commenting on social media, serving as customer service representatives, and more.^21^ Understanding their direct and indirect impacts on human behavior and connections is imperative to leveraging their utility without sacrificing our humanity.

### Limitations of the study

Our studies explored how a relatively simple machine (i.e., a computer program represented by an image of a robot on participants’ screens) impacted human behavior. Future work should consider how more advanced AI may alter the effects we document here. For example, AI that can verbally communicate, such as ChatGPT or Gemini, may yield different effects,^66^ as communication has long been demonstrated to promote cooperation.^67^^,^^68^^,^^69^ Work that incorporates AI of varying degrees of complexity (e.g., bots with the ability to learn or adapt) may highlight how different kinds of bot behaviors affect human cooperative behaviors. And robots that are increasingly humanoid in appearance, or display human-like emotions—especially if they interact with participants face-to-face, rather than online as in our studies—might feel more realistic or “human-like” to their human interaction partners^70^; in turn, they may be more likely to encourage empathic concern and perspective-taking, and promote cooperation, at levels more closely approximating cooperation with humans.^71^^,^^72^

Additionally, we did not measure empathic concern and perspective-taking prior to exposing participants to our manipulation (for example, by giving the Interpersonal Reactivity Index as a measure of trait empathy^46^ at a time 1^42^^,^^73^ before informing participants that they were partnered with a bot or ostensible human). Nor did we measure or assess the effects of other potentially relevant personality traits (e.g., agreeableness or social value orientation) or characteristics (e.g., cultural background or prior experience with AI). Measuring these at the beginning of future work in this area would allow for an exploration of whether these differences alter our findings. For example, people with high trait empathy or from different cultural backgrounds might not show the negative emotional spillover effects of interaction with bots. Qualitative analyses of open-ended questions (which we did not include in our studies) would also give deeper insight into the emotional or cognitive mechanisms driving the effects we reported here.

Finally, the current studies do not allow us to rule out other possible pathways by which interactions with a bot spill over to interactions with humans. While Study 2 allows us to rule out that suspicion differences were causing differences in cooperation patterns, we cannot rule that out in Study 1. Additionally, while our proposed spillover pathways (behavioral spillover and emotional spillover) were well-grounded in prior literature, they are neither mutually exclusive to each other, nor to other possibilities. For example, interactions with a bot compared to an (ostensible) human may alter people’s risk perceptions– if bots are seen as more predictable, even though both players were pre-programmed, it may make the change to a human partner confusing and unpredictable, reducing cooperation, compared to those participants who went from one (ostensible) human to another human partner. More generally, understanding how interactions with AI impact the humans’ treatment of other humans is critical as people increasingly interact with AI in daily life.

## Resource availability

### Lead contact

Further information and requests for resources should be directed to and will be fulfilled by the lead contact, Ashley Harrell (ashley.l.harrell@duke.edu).

### Materials availability

This study did not generate new unique materials.

### Data and code availability

• The data for both studies have been deposited on the Open Science Framework: https://osf.io/z95ev/.
• Code for replicating the results from both studies is available on the Open Science Framework: https://osf.io/z95ev/.
• Any other resources required for the reproduction of the article are available by request.

## Acknowledgments

We thank Loey Allen, Gabi Carter, Sua Cho, Luke Flyer, and Sydney Maynor for valuable research assistance. The Department of Sociology and Trinity College at 10.13039/100006510Duke University and the Department of Information Technology, Analytics, and Operations at the 10.13039/100008109University of Notre Dame and the 10.13039/100028931Kemper Foundation funded the research.

## Author contributions

Authors contributed equally. Conceptualization and study design: A.H. and M.T. Programming and data collection supervision: A.H. Data analysis: A.H. and M.T. Wrote the article: A.H. and M.T.

## Declaration of interests

The authors declare no competing interests.

## STAR★Methods

### Key resources table

**Table:** 

REAGENT or RESOURCE	SOURCE	IDENTIFIER
**Deposited data**		

Study 1 Data	OSF Registry	https://osf.io/z95ev/
Study 2 Data	OSF Registry	https://osf.io/z95ev/
R Script	OSF Registry	https://osf.io/z95ev/

**Software and algorithms**		

SMARTRIQS	Molnar, 2019	https://doi.org/10.1016/j.jbef.2019.03.005
R v4.3.2	R Core Team	https://www.r-project.org

**Other**		

Preregistration for Study 1	AsPredicted	https://aspredicted.org/CTF_KCV
Preregistration for Study 2	AsPredicted	https://aspredicted.org/KX2_DNY
Experimental Instructions	OSF Registry	https://osf.io/z95ev/

### Experimental model and study participant details

Both studies took place entirely online and were programmed using SMARTRIQS.^74^ Participants were recruited via Prolific.com^75^^,^^76^ for a 15-min study (the average completion time was 9.63 and 12.75 min for Study 1 and 2 respectively), in exchange for a flat payment of $3 plus the chance to receive a bonus ranging from $1-$4. They were required to be currently residing in the United States and have an approval rating on Prolific of at least 98%. Study 1 participants were prevented from participating in Study 2. The study design (and, for Study 2, an amendment to the protocol for Study 1) was approved by the Institutional Review Boards of Duke University and the University of Notre Dame. Informed consent was obtained from all participants prior to their participation.

We pre-registered our research questions, conditions, sample size, and data exclusions (Study 1: https://aspredicted.org/CTF_KCV, Study 2: https://aspredicted.org/KX2_DNY) before data collection began. We conducted rough power analyses via simulations on a hypothetical generalized linear multilevel model and, as described in our pre-registrations, aimed to recruit 2,000 participants in each study. Our final sample size for Study 1 was 2053 participants; for Study 2 it was 2118. Participants were randomly assigned to experimental conditions in both studies, as described in more detail in the next section.

We obtained participants’ age and gender, but no other demographic variables, from Prolific; the supplemental information contains models showing whether and how these demographics predicted our outcome variables or interacted with condition to predict the outcomes; see also Table S1. For Study 1, the average age of our participants was 39.1 years; 54.8% were male. For Study 2, the average age was 38.9; 46.6% were male. For Study 1, to facilitate pairing participants with a same-condition alter in the spillover phase (as described in more detail in the main text), participants were randomly assigned to experimental conditions was randomly assigned (via a random number generator) at the session level. We ran sessions for approximately 50 participants at a time in October and November 2023. For Study 2, participants were individually randomly assigned to experimental condition via the study program. We ran study sessions of approximately 50 participants at a time in January and February 2024.

### Method details

Across both studies, after a consent screen was a set of instructions for a standard version of a repeated PDG.^77^^,^^78^^,^^79^ Participants were told that they would make decisions in a series of rounds with another player, and that their and the other’s decisions in one randomly chosen round^80^^,^^81^ would determine their bonus. We manipulated whether they were told in advance that the other player with whom they would be paired was a robot (those randomly assigned to the *bot* condition) or another participant (the *human* condition). Participants in the bot condition were told nothing about what would happen to the bot’s earnings, in order to keep differences in the instructions (which, in the human condition, also did not further mention others' earnings) as similar as possible. Prior work has demonstrated that this strategy, as well as strategies where a third-party participant receives the money that a bot earns, are each associated with reduced cooperation with bots (compared to cooperation with other humans).^82^ Thus, while any differences in cooperation with bots versus humans in the manipulation phase may partly be due to the perception that bots don’t need or want money or that their earnings will go to “waste”, they cannot fully explain the differences.^39^

As in a standard PDG, there was a tension between what was best for a given player and what was best for the collective. The best payoff was obtained from unilateral defection (choosing “Purple”, while the other chose “Orange”, in the study instructions), mutual decisions to cooperate followed, and unilateral cooperation resulted in the lowest payoff. After learning what the other player chose and their payoffs for the round, the instructions explained, they would proceed to another round with the same partner. A series of quiz questions to check their understanding of how the task worked followed, including a manipulation check asking whether they would be paired with a bot or human during the task.

Study 1 participants began the decision-making task after the quiz. While it was not announced in advance, participants completed ten rounds of what we call the *manipulation phase*. Regardless of condition, in this phase participants were paired with a pre-programmed partner. We manipulated whether the bot or ostensible other participant made choices according to several strategies. In the *always cooperate* and *always defect* conditions, the pre-programmed alter cooperated or defected, respectively, in every round of the manipulation phase. In the *tit for tat* condition, the alter cooperated during the first round and mimicked the focal participant’s previous-round behavior in all subsequent rounds.^77^ Finally, we included a *random* condition as a baseline, where the other player’s decisions were made at random. This minor deception^49^^,^^50^^,^^51^ allowed us to hold constant the manipulation phase alter’s behavior across conditions, and therefore assess the effect of the first alter’s type (human or bot) on subsequent behavior with a human partner, rather than the effect of any difference in decisions made by a computer program versus a real other participant’s behavior.

After the ten rounds of the manipulation phase, a new set of instructions told participants that they would be paired with a new other player for another series of rounds. Across all conditions, participants were told (correctly) that their new alter would be another participant. They answered an additional quiz question to ensure that they understood the new instructions before beginning the second phase. This second phase is where we planned to assess spillover effects based on the manipulations in the manipulation phase; we therefore call this the *spillover phase*.

In the spillover phase of Study 1, participants were paired with another participant who had previously completed their same condition (and thus, had the same experience as they had had in the manipulation phase). Occasionally (specifically, for 4.4% of our sample in Study 1 and 4.0% for Study 2), another participant in the participant’s same condition was not available after waiting for three minutes. When that happened, the screen progressed as if another participant had become available; the “other participant” was programmed to play Tit for Tat. Additionally, if a participant went idle in a round by not making a decision in more than two minutes, the computer filled in for that round by playing Tit for Tat so that the study could progress and end on time. 1.6% of Study 1 participants (and 1.6% of Study 2 participants) had a partner who went idle in one or more rounds; any programmed decisions made on behalf of idle participants were not included in our analyses. Participants were never aware that they had been paired with the computer or that the computer had filled in for their idle partner for the round.

After proceeding for another ten rounds in the spillover phase, the decision-making task was complete. Participants answered a brief open-ended question about what they thought the study was about and two questions about their perceptions of whether the partner with whom they were paired in the first and second set of rounds, respectively, was a real person or not. Our aim with this question was to assess whether participants in the *bot* condition were more suspicious that their spillover phase partner was an actual other participant, compared to those in the *human* condition, and if so, whether this could explain why those in the bot condition were less cooperative in the spillover phase. See the supplemental information for more discussion and our analyses on these items, including tests showing that suspicion of the spillover phase partner did not differ by condition in Study 2, and that our effects are robust after controlling for suspicion in both studies.

Finally, participants were debriefed about the purpose of the study, including the use of deception about the identity of the manipulation phase partner for those in the *human* condition and why it was needed, in line with best-practice recommendations and prior work showing that being deceived in one study does not affect the validity of others.^52^^,^^53^^,^^54^ On the debrief screen (see the supplemental information for the full text of the debriefing statement), we also told participants that they would be paid based on one randomly selected round from those rounds where they were paired with an actual other participant.

Following a power analysis described in our pre-registration, we aimed to collect approximately 2,000 complete responses in Study 1. After omitting 114 responses for reasons we pre-registered (duplicate IP addresses, *n* = 1, answering fewer than three of our six quiz questions incorrectly, *n* = 26, and invalid or suspicious answers to our open-ended question, *n* = 87), we retained 2,053 participants for analysis.

The procedure for Study 2 was virtually identical to Study 1, with two exceptions. First, in Study 1 we paired participants in the spillover phase so participants were matched based on their condition in the manipulation phase. Spillover effects in Study 1, then, may have been reinforced or magnified by the spillover phase partner’s behavior, which had been affected by the same previous experience that the focal participant had. For a more conservative test of spillover effects, in Study 2 we paired participants without regard to their manipulation phase condition.

Second, Study 2 included several mediation items to assess how interactions with bots (vs. humans) impacted downstream cooperation. Specifically, participants completed a scale measuring *empathic concern* toward their partner and a scale measuring *perspective-taking* of their partner,^41^ after reading the instructions (and thus, finding out that their upcoming partner was either a bot or a human), but before the phase of decision-making rounds began, for both the manipulation phase and the spillover phase. The supplemental information contains text from the study instructions, including the scale items, which we averaged to create a single item for total empathy toward each partner (because perspective-taking and empathic concern as separate sub-scales were highly correlated, *r* = 0.53. Following past work, we combined them into one *total empathy toward the partner* scale^62^). After omitting 138 responses for reasons we pre-registered (answering fewer than three of our six quiz questions incorrectly, *n* = 17, and invalid or suspicious answers to our open-ended question, *n* = 121), we retained 2,118 participants for analysis.

### Quantification and statistical analysis

We conducted all statistical analyses in R. All analyses are contained within the code scripts on OSF (https://osf.io/z95ev/). We describe the statistical tests, significance levels, sample sizes, and variables used in the main text, the figures and figure legends, and the table and table legends. All reported *p*-values are two-tailed.
