# Supplementary material for: Evidence of spillovers from (non)cooperative human-bot to human-human interactions
Source: iScience. 2025 Jun 25;28(8):113006. doi: 10.1016/j.isci.2025.113006 (PMC12355112; doi:10.1016/j.isci.2025.113006)
Supplement: Document S1. Figures S1 and S2 and Tables S1–S16 [file mmc1.pdf]

iScience, Volume *28*

Supplemental information

Evidence of spillovers from (non)cooperative human-bot to human-human interactions

Ashley Harrell and Margaret L. Traeger

Supplemental Information

Contents

Supplemental Tables and Figures

Figure S1. Participant cooperation behavior patterns by condition and phase, Study 1.

Figure S2. Participant cooperation behavior patterns by condition and phase, Study 2.

Table S1. Sample demographics, Studies 1 and 2.

Table S2. *Manipulation phase* models discussed in main text, Study 1.

Table S3. Hazard of defection by condition in the manipulation phase and spillover phase, Study 1.

Table S4. Interactions with gender, age, and being paired with a bot, *Manipulation phase*, Study 1.

Table S5. *Spillover phase* models discussed in main text, Study 1.

Table S6. Interactions with gender, age, and being paired with a bot, *Spillover phase*, Study 1.

Table S7. Rating of the partner as “a real person”, for both the manipulation phase and spillover phase partner, Study 1.

Table S8. Rating of the partner as “a real person”, for both the manipulation phase and spillover phase partner, with interactions between conditions, Study 1.

Table S9. *Manipulation phase* models discussed in main text, Study 2.

Table S10. Hazard of defection by condition in the manipulation phase and spillover phase, Study 2.

Table S11. Interactions with gender, age, and being paired with a bot, *Manipulation phase*, Study 2.

Table S12. *Spillover phase* models discussed in main text, Study 2.

Table S13. Interactions with gender, age, and being paired with a bot, *Spillover phase*, Study 2.

Table S14. Rating of the partner as “a real person”, for both the manipulation phase and spillover phase partner, Study 2.

Table S15. Rating of the partner as “a real person”, for both the manipulation phase and spillover phase partner, with interactions between conditions, Study 2.

Table S16. Mediation analysis: the effect of being paired with a bot in the manipulation phase on empathic concern, perspective taking, and cooperation rate in the spillover phase in Study 2.

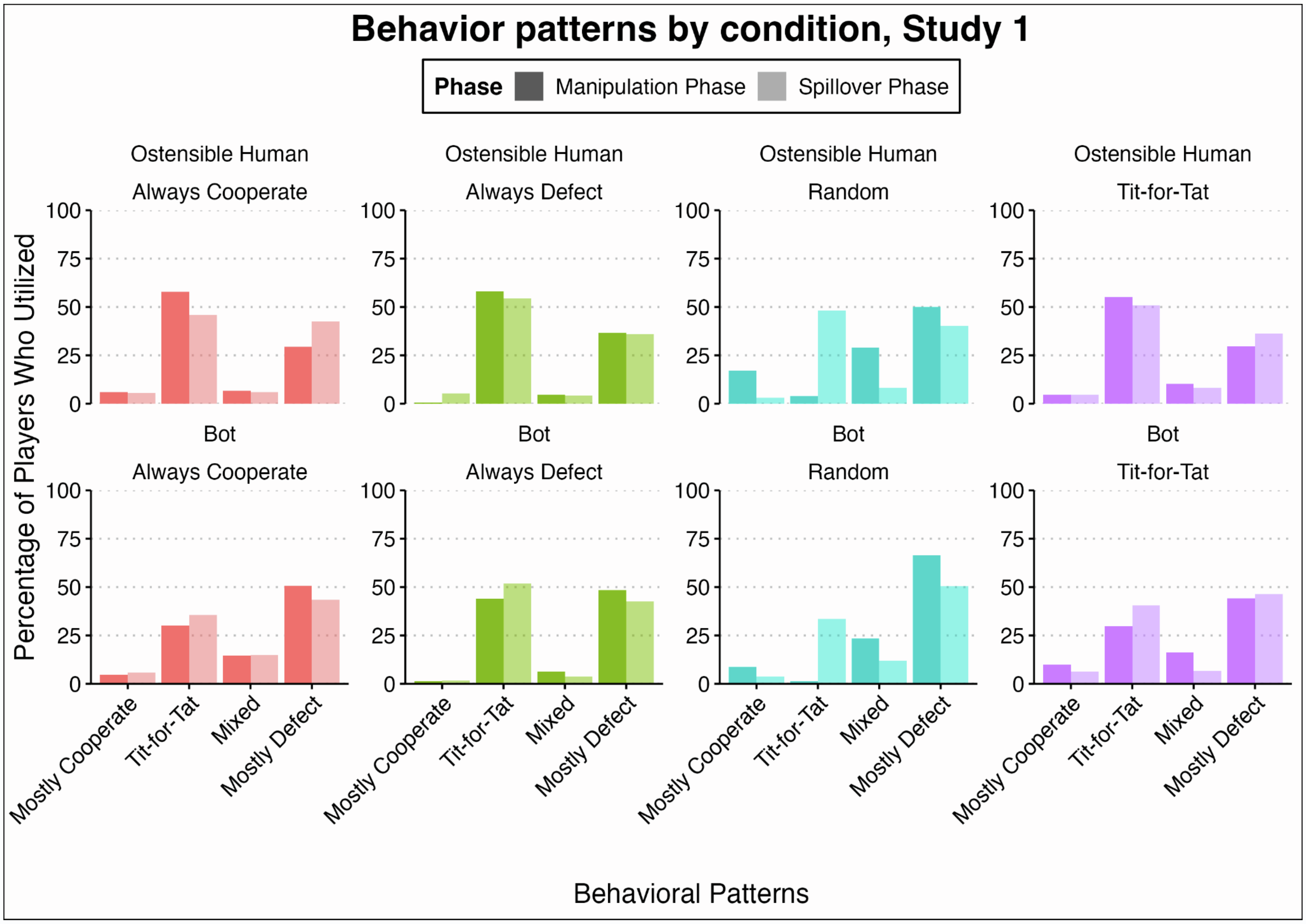

Figure S1. Participant cooperation behavior patterns by condition and phase, Study 1, related to Figure 2. Percentage of participants within an experimental condition who mostly cooperate, play Tit for Tat (TFT)[i], cooperate half the time and defect half the time, or mostly defect.[ii] In the manipulation phase (solid bars), TFT was the most common strategy toward ostensible human partners (top row) that played any strategy except random, where participants tended to mostly defect. When the manipulation phase partner was a bot (bottom row), participants commonly engaged in "mostly defect" strategies, even when the (bot) partner always cooperated or played TFT. They were also highly likely to play TFT against an always-defecting bot partner (which also would have led to high levels of defection, i.e., defection in all rounds except the first). In the spillover phase (transparent bars), participants who had previously been paired with an ostensible human continued to most commonly play TFT with their new human partner, regardless of the previous partner's strategy. Those who had previously been paired with a bot typically engaged in either TFT or mostly-defect behavior, even with the new human partner.

---

[i] There are, of course, patterned strategies other than Tit for Tat. For example, Grim Trigger involves ego cooperating until their alter defects; then, ego switches to defection for all remaining rounds. However, in our study, this strategy is conflated with playing Tit for Tat when ego's pre-programmed partner in the manipulation phase was randomly assigned to always defect (i.e., Tit for Tat would also lead to a "start with cooperation, then switch to defection for all remaining rounds" strategy against an always-defecting partner). Likewise, we did not assess a distinct "always cooperate" or "always defect" strategy because an "always cooperate" strategy is conflated with ego playing Tit for Tat when their pre-programmed partner was assigned to always cooperate or play Tit for Tat. Therefore, we assessed the mutually exclusive sequences of whether participants played Tit for Tat with their partner and, if they did not, whether they tended toward cooperation ("mostly cooperate")[i] or defection ("mostly defect"), or an equal mix.

[ii] The "mostly cooperate" strategy also includes those participants who *always* cooperated against a partner who was not pre-programmed to play Tit for Tat or always cooperate (for instance, if ego always cooperated against a pre-programmed partner who made choices at random or, in the spillover phase, if ego always cooperated with a partner who played a mixed strategy). However, participants who always cooperated against a pre-programmed partner who played Tit for Tat or always cooperate (or a real other partner who always cooperated) were categorized as having played Tit for Tat.

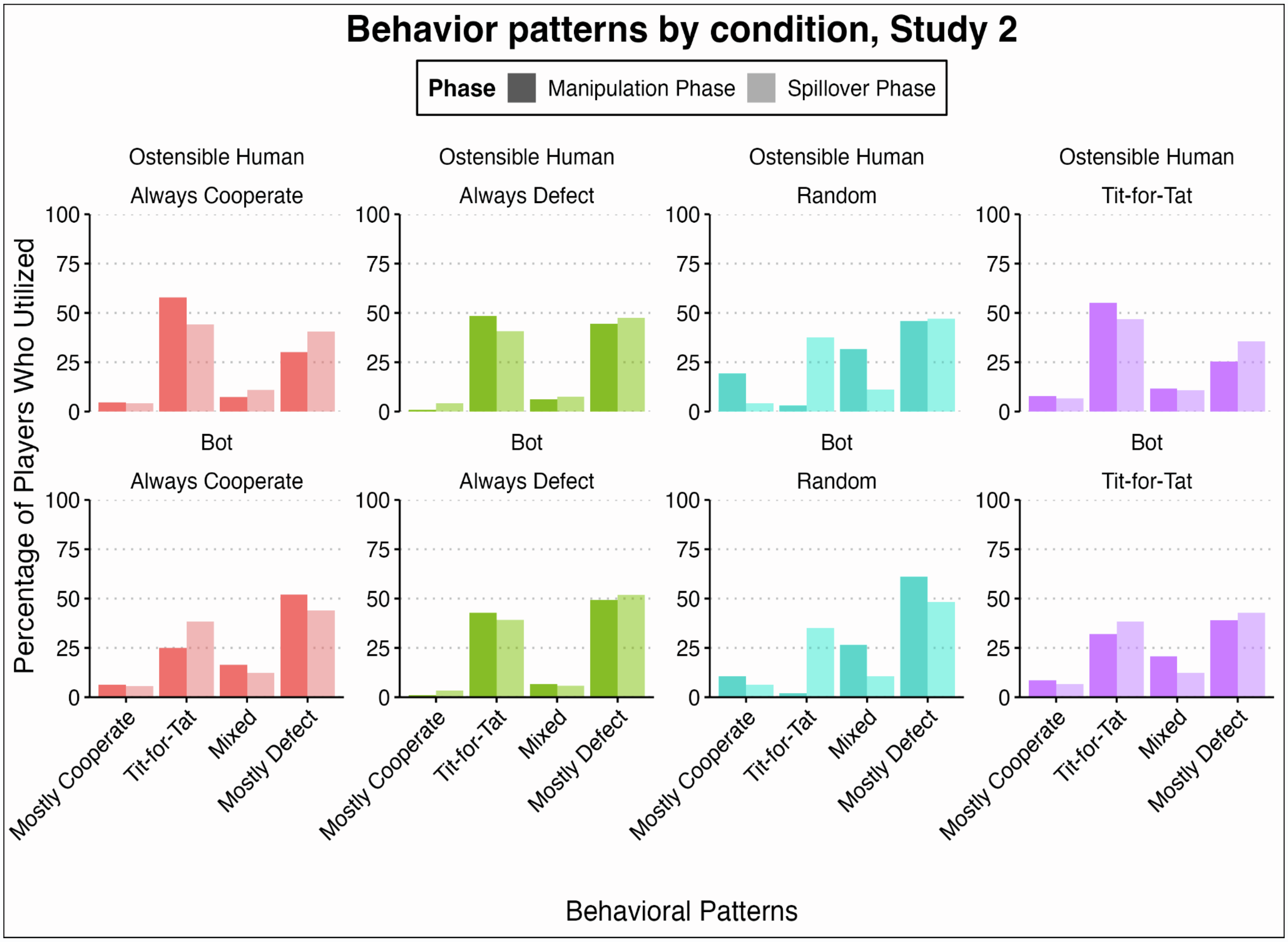

Figure S2. Participant cooperation behavior patterns by condition and phase, Study 2, related to Figure 3. Percentage of participants within an experimental condition who mostly cooperate, play Tit for Tat, cooperate half the time and defect half the time, or mostly defect. In the manipulation phase, Tit for Tat was the most common strategy toward ostensible human partners (top row) that played any strategy except random (where participants mostly defect toward their partner, as in Study 1). When the manipulation phase partner was a bot (bottom row), participants engaged in mostly defect strategies; as in Study 1, there they were also highly likely to play Tit for Tat against an always-defecting bot partner (which also would have led to high levels of defection, i.e., defection in all rounds except the first). In the spillover phase (transparent bars), participants who had previously been paired with an ostensible human who always cooperated or played Tit for Tat continued to most commonly play Tit for Tat with their new human partner; defection was higher if the previous partner had always defected or played at random. For those who had previously been paired with a bot, regardless of condition, participants were most likely to use a mostly defect strategy in the subsequent phase.

		Study 1	Study 2
**$N_{participants}$**		2,053	2,118
**Age (mean (SD))**		39.1 (12.0)	38.9 (12.8)
	Missing (N)	35	13
**Sex (%)**	*Female*	909 (44.3)	1,115 (52.6)
	Male	1,126 (54.8)	988 (46.6)
	Prefer not to say	5 (0.2)	4 (0.2)
	Missing	13 (0.6)	11 (0.5)

Table S1. Sample demographics, Studies 1 and 2, related to STAR Methods. All participants in both studies were required (via Prolific) to be currently residing in the United States and have an approval rating on Prolific of at least 98%. Participants who completed Study 1 were not able to complete Study 2. We viewed one reported age of 99 (in Study 1) and two reported ages of 104 (in Study 2) as outliers and re-coded them as missing.

	Model 1			Model 2		
Predictors	*OR*	*SE*	*Bootstrapped 95% CI*	*OR*	*SE*	*Bootstrapped 95% CI*
Intercept	1.83 **	0.35	1.24 – 2.67	1.83 **	0.39	1.22 – 2.88
Partner played Tit for Tat (TFT)	2.53 **	0.76	1.40 – 4.54	2.56 **	0.79	1.46 – 4.28
Partner always cooperated (C)	2.34 **	0.69	1.33 – 4.29	2.39 **	0.72	1.36 – 4.07
Partner always defected (D)	0.56 *	0.15	0.34 – 0.99	0.58 *	0.16	0.34 – 1.02
Partner was a bot (Bot)	0.35 ***	0.09	0.21 – 0.60	0.35 ***	0.09	0.20 – 0.60
Round in the phase (R)	0.61 ***	0.03	0.55 – 0.67	0.60 ***	0.03	0.54 – 0.66
TFT x R	2.03 ***	0.15	1.85 – 2.49	2.06 ***	0.15	1.89 – 2.50
C x R	2.01 ***	0.14	1.82 – 2.42	2.05 ***	0.15	1.87 – 2.47
D x R	0.46 ***	0.04	0.38 – 0.53	0.46 ***	0.04	0.39 – 0.54
Bot x R	1.01	0.06	0.88 – 1.14	1.03	0.06	0.88 – 1.16
TFT x Bot	0.54	0.21	0.25 – 1.13	0.52	0.21	0.24 – 1.23
C x Bot	0.64	0.25	0.30 – 1.39	0.64	0.26	0.32 – 1.41
D x Bot	1.38	0.51	0.65 – 2.80	1.36	0.52	0.64 – 2.91
TFT x Bot x R	0.87	0.08	0.71 – 1.00	0.85	0.08	0.70 –0.99
C x Bot x R	0.69 ***	0.06	0.56 – 0.81	0.67 ***	0.06	0.54 – 0.78
D x Bot x R	1.62 ***	0.16	1.32 – 2.04	1.59 ***	0.16	1.32 – 1.98
Age (standardized)				1.05	0.07	0.92 – 1.20
Male				0.98	0.14	0.75 – 1.28
Observations	20530			20090		
Marginal R^2 / Conditional R^2	0.345 / 0.893			0.346 / 0.894		

** p<0.05   ** p<0.01   *** p<0.001*

Table S2. *Manipulation phase* models discussed in main text, Study 1, related to Figure 2. Cooperation decisions made in the *manipulation phase* (i.e., the first phase) of Study 1. Logistic generalized linear models with random intercepts at the participant level and random slopes for round in the phase. *Round in the phase* ranges from 0 (round 1) to 9 (round 10). We computed bootstrapped confidence intervals using the percentile bootstrap method, with N = 1,000 resamples. Model 2 contains control terms for participant demographics. 44 participants were dropped from this model for missingness on age and/or gender (see Table S1).

	Model 1 Manipulation phase			Model 2 Spillover phase		
Predictors	*HR*	*SE*	*Bootstrapped 95% CI*	*HR*	*SE*	*Bootstrapped 95% CI*
Manipulation phase partner (MPP) played Tit for Tat (TFT)	0.47 ***	0.04	0.41 – 0.55	0.58 ***	0.05	0.49 – 0.69
MPP always cooperated (C)	0.49 ***	0.04	0.42 – 0.56	0.75 *	0.06	0.62 – 0.85
MPP always defected (D)	1.35 ***	0.09	1.23 – 1.48	1.09	0.09	0.92 – 1.22
MPP was a bot (B)	1.52 ***	0.08	1.39 – 1.66	1.55 ***	0.09	1.37 – 1.72
Observations	2053			2053		
R^2 Nagelkerke	0.160			0.702		

* *p<0.05* ** *p<0.01* *** *p<0.001*

Table S3. Hazard of defection by condition in the manipulation phase and spillover phase, Study 1, related to Figure 2. Time to first defection by experimental condition using Cox proportional hazards models. Model 2 includes a frailty term to account for participants belonging to a dyad with a real other participant (this was not necessary in Model 1 because the alter in the manipulation phase was a pre-programmed bot or ostensible human). The variance ($\sigma^2 = 1.35$) of the frailty term was significant ($p < 0.001$). We computed bootstrapped confidence intervals using the percentile bootstrap method, with N = 1,000 resamples.

	Model 1: Gender			Model 2: Age		
Predictors	*OR*	*SE*	*Bootstrapped 95% CI*	*OR*	*SE*	*Bootstrapped 95% CI*
Intercept	1.59 *	0.35	1.03 – 2.49	1.84 **	0.39	1.20 – 2.85
Partner played Tit for Tat (TFT)	2.51 **	0.77	1.44 – 4.28	2.54 **	0.78	1.44 – 4.13
Partner always cooperated (C)	2.36 **	0.71	1.32 – 4.02	2.37 **	0.72	1.33 – 3.98
Partner always defected (D)	0.57 *	0.15	0.33 – 0.98	0.57 *	0.15	0.34 – 1.00
Partner was a bot (Bot)	0.45 **	0.14	0.24 – 0.80	0.35 ***	0.09	0.20 – 0.60
Round in the phase (R)	0.63 ***	0.03	0.56 – 0.70	0.60 ***	0.03	0.54 – 0.66
Age (standardized)	1.06	0.07	0.93 – 1.20	0.96	0.10	0.78 – 1.18
Male (ref: Female)	1.31	0.27	0.90 – 1.92	0.97	0.14	0.74 – 1.27
TFT x R	2.08 ***	0.15	1.90 – 2.54	2.06 ***	0.15	1.88 – 2.49
C x R	2.07 ***	0.15	1.88 – 2.49	2.05 ***	0.15	1.87 – 2.47
D x R	0.46 ***	0.04	0.39 – 0.55	0.46 ***	0.04	0.39 – 0.54
Bot x R	1.00	0.07	0.85 – 1.15	1.03	0.06	0.88 – 1.16
TFT x Bot	0.54	0.22	0.25 – 1.21	0.53	0.21	0.25 – 1.16
C x Bot	0.66	0.27	0.33 – 1.45	0.65	0.26	0.33 – 1.44
D x Bot	1.40	0.53	0.66 – 3.07	1.37	0.52	0.66 – 2.94
TFT x Bot x R	0.85	0.08	0.69 –0.98	0.86	0.08	0.70 – 0.99
C x Bot x R	0.67 ***	0.06	0.54 – 0.78	0.68 ***	0.06	0.55 – 0.78
D x Bot x R	1.58 ***	0.16	1.30 – 1.95	1.59 ***	0.16	1.31 – 1.98
Male x R	0.91	0.05	0.83 – 1.01			
Male x Bot	0.60	0.17	0.35 – 1.00			
Male x Bot x R	1.08	0.07	0.94 – 1.22			
Age x R				0.98	0.03	0.93 – 1.03
Age x Bot				1.18	0.16	0.91 – 1.57
Age x Bot x R				1.04	0.03	0.97 – 1.12
Observations	20090			20090		
Marginal R^2 / Conditional R^2	0.346 / 0.894			0.347 / 0.894		

* *p<0.05* ** *p<0.01* *** *p<0.001*

Table S4. Interactions with gender, age, and being paired with a bot, *Manipulation phase*, Study 1, related to Figure 2. Logistic generalized linear models with random intercepts at the participant level and random slopes for round in the phase. *Round in the phase* ranges from 0 (round 1) to 9 (round 10). We computed bootstrapped confidence intervals using the percentile bootstrap method, with N = 1,000 resamples. 44 participants were dropped from this model for missingness on age and/or gender (see Table S1).

	Model 1			Model 2		
Predictors	*OR*	*SE*	*Bootstrapped 95% CI*	*OR*	*SE*	*Bootstrapped 95% CI*
Intercept	0.66 *	0.11	0.49 – 0.90	0.48 ***	0.10	0.34 – 0.70
Manipulation phase partner (MPP) played Tit for Tat (TFT)	3.11 ***	0.64	2.09 – 4.56	2.96 ***	0.61	2.04 – 4.17
MPP always cooperated (C)	1.86 **	0.37	1.24 – 2.66	1.85 **	0.38	1.25 – 2.65
MPP always defected (D)	0.94	0.19	0.66 – 1.36	0.95	0.19	0.67 – 1.37
MPP was a bot (Bot)	0.48 ***	0.07	0.37 – 0.64	0.58 ***	0.09	0.45 – 0.81
Round in the phase (R)	0.77 ***	0.06	0.67 – 0.89	0.76 ***	0.06	0.67 – 0.89
Bot x R	0.82 **	0.05	0.70 – 0.90	0.82 **	0.05	0.70 – 0.91
TFT x R	1.20 *	0.11	1.03 – 1.48	1.21 *	0.11	1.01 – 1.45
C x R	0.94	0.08	0.80 – 1.14	0.93	0.08	0.79 – 1.13
D x R	0.66 ***	0.06	0.55 – 0.80	0.66 ***	0.06	0.54 – 0.79
Cooperated this round of the manipulation phase	3.60 ***	0.25	3.05 – 4.01	3.62 ***	0.25	3.09 – 4.10
Age (standardized)				1.05	0.05	0.98 – 1.14
Male				1.23 *	0.11	1.05 – 1.43
Rating of the manipulation phase partner as “a real person”				1.08 **	0.03	1.02 – 1.13
Rating of the spillover phase partner as “a real person”				0.98	0.02	0.94 – 1.02
Observations	20523			20083		
Marginal R^2 / Conditional R^2	0.123 / 0.930			0.124 / 0.931		

* *p<0.05* ** *p<0.01* *** *p<0.001*

Table S5. *Spillover phase* models discussed in main text, Study 1, related to Figure 2. Cooperation decisions made in the *spillover phase* (i.e., the second phase) of Study 1, based on the manipulations that occurred in the *manipulations phase* (i.e., the first phase). Logistic generalized linear models with random intercepts at the dyad and participant level and random slopes for round in the phase. *Round in the phase* ranges from 0 (round 1) to 9 (round 10). We computed bootstrapped confidence intervals using the percentile bootstrap method, with N = 1,000 resamples. Model 2 contains control terms for participant demographics, and suspicion of the manipulation and spillover phase partners. 44 participants were dropped from this model for missingness on age and/or gender (see Table S1).

	Model 1: Gender			Model 2: Age		
Predictors	*OR*	*SE*	*Bootstrapped 95% CI*	*OR*	*SE*	*Bootstrapped 95% CI*
Intercept	0.59 **	0.11	0.42 – 0.83	0.59 **	0.10	0.44 – 0.81
Manipulation phase partner (MPP) played Tit for Tat (TFT)	2.97 ***	0.62	2.07 – 4.22	2.97 ***	0.62	2.04 – 4.24
MPP always cooperated (C)	1.81 **	0.37	1.24 – 2.62	1.82 **	0.37	1.26 – 2.63
MPP always defected (D)	0.94	0.19	0.67 – 1.40	0.94	0.19	0.67 – 1.37
MPP was a bot (Bot)	0.47 ***	0.09	0.34 – 0.67	0.50 ***	0.07	0.39 – 0.67
Round in the phase (R)	0.80 **	0.06	0.69 – 0.94	0.77 ***	0.06	0.67 – 0.88
Cooperated this round of the manipulation phase	3.65 ***	0.25	3.12 – 4.12	3.64 ***	0.25	3.10 – 4.11
Male (ref: Female)	1.23	0.19	0.97 – 1.59	1.21 *	0.11	1.04 – 1.41
Age (standardized)	1.06	0.05	0.98 – 1.14	0.98	0.07	0.87 – 1.12
Bot x R	0.78 **	0.06	0.67 – 0.88	0.82 **	0.05	0.70 – 0.91
TFT x R	1.22 *	0.11	1.05 – 1.52	1.22 *	0.11	1.05 – 1.51
C x R	0.93	0.09	0.79 – 1.13	0.93	0.09	0.80 – 1.13
D x R	0.66 ***	0.06	0.54 – 0.79	0.66 ***	0.06	0.54 – 0.79
Male x Bot	1.10	0.22	0.74 – 1.55			
Male x R	0.91	0.05	0.83 – 1.00			
Male x Bot x R	1.09	0.08	0.96 – 1.23			
Age x Bot				1.06	0.10	0.88 – 1.27
Age x R				1.05	0.03	0.99 – 1.10
Age x Bot x R				0.97	0.03	0.91 – 1.04
Observations	20083			20083		
Marginal R^2 / Conditional R^2	0.124 / 0.931			0.124 / 0.931		

** p<0.05   ** p<0.01   *** p<0.001*

Table S6. Interactions with gender, age, and being paired with a bot, *Spillover phase*, Study 1, related to Figure 2. Logistic generalized linear models with random intercepts at the dyad and participant level and random slopes for round in the phase. *Round in the phase* ranges from 0 (round 1) to 9 (round 10). We computed bootstrapped confidence intervals using the percentile bootstrap method, with N = 1,000 resamples. 44 participants were dropped from this model for missingness on age and/or gender (see Table S1).

	Model 1: Ratings of the *manipulation phase partner* (a bot or ostensible human			Model 2: Ratings of the *spillover phase partner* (a real other participant		
Predictors	*Est.*	*SE*	*Bootstrapped 95% CI*	*Est.*	*SE*	*Bootstrapped 95% CI*
Intercept	3.98***	0.09	3.79 – 4.17	4.29***	0.11	4.05 – 4.51
Manipulation phase partner (MPP) was a bot	-2.38***	0.08	-2.54 – -2.23	-0.29**	0.10	-0.47 – -0.10
MPP played Tit for Tat	0.21	0.11	0.00 – 0.41	0.04	0.13	-0.23 – 0.31
MPP always defected	-0.04	0.11	-0.27 – 0.16	-0.21	0.13	-0.50 – 0.05
MPP always cooperated	-0.17	0.11	-0.39 – 0.03	0.03	0.14	-0.23 – 0.30
Observations	2053			2053		
R^2 / R^2 adjusted	0.314 / 0.312			0.006 / 0.005		

Table S7. Rating of the partner as “a real person”, for both the manipulation phase and spillover phase partner, Study 1, related to STAR Methods. Linear regressions of participants’ suspicion (lower values indicate greater suspicion/less rating the partner as a real person) of their manipulation and spillover phase partners. We computed bootstrapped confidence intervals using the percentile bootstrap method, with N = 1,000 resamples.

	Model 1: Ratings of the *manipulation phase partner* (a bot or ostensible human			Model 2: Ratings of the *spillover phase partner* (a real other participant		
Predictors	*Est.*	*SE*	*Bootstrapped 95% CI*	*Est.*	*SE*	*Bootstrapped 95% CI*
Intercept	3.97***	0.12	3.67 – 4.27	4.27***	0.14	3.99 - 4.56
Manipulation phase partner (MPP) was a bot (Bot)	-2.37***	0.16	-2.69 – -2.02	-0.26	0.19	-0.64 - 0.12
MPP played Tit for Tat (TFT)	0.47***	0.16	0.07 – 0.88	0.07	0.20	-0.32 - 0.48
MPP always defected (D)	-0.12	0.16	-0.50 – 0.26	-0.04	0.19	-0.43 - 0.33
MPP always cooperated (C)	-0.30	0.16	-0.71 – 0.09	-0.13	0.20	-0.51 - 0.27
Bot x TFT	-0.49*	0.22	-0.95 – -0.04	-0.05	0.27	-0.58 - 0.48
Bot x D	0.17	0.22	-0.28 – 0.61	-0.38	0.27	-0.89 - 0.16
Bot x C	0.26	0.22	-0.18 – 0.72	0.32	0.27	-0.23 - 0.85
Observations	2053			2053		
R^2 / R^2 adjusted	0.318 / 0.316			0.010 / 0.006		

Table S8. Rating of the partner as "a real person", for both the manipulation phase and spillover phase partner, with interactions between conditions, Study 1, related to STAR Methods. Linear regressions of participants' suspicion (lower values indicate greater suspicion/less rating the partner as a real person) of their manipulation and spillover phase partners. We computed bootstrapped confidence intervals using the percentile bootstrap method, with N = 1,000 resamples. See Table S7 for models with just the main effects.

	Model 1			Model 2		
Predictors	*OR*	*SE*	*Bootstrapped 95% CI*	*OR*	*SE*	*Bootstrapped 95% CI*
Intercept	1.10	0.18	0.82 – 1.53	1.28	0.22	0.92 – 1.74
Partner played Tit for Tat (TFT)	3.99 ***	1.00	2.33 – 6.20	4.15 ***	1.05	2.51 – 6.55
Partner always cooperated (C)	2.62 ***	0.66	1.58 – 4.09	2.58 ***	0.66	1.60 – 4.16
Partner always defected (D)	0.88	0.21	0.55 – 1.39	0.88	0.21	0.56 – 1.49
Partner was a bot (Bot)	0.52 **	0.12	0.34 – 0.83	0.52 **	0.12	0.34 – 0.84
Round in the phase (R)	0.79 ***	0.03	0.73 – 0.84	0.79 ***	0.03	0.73 – 0.84
TFT x R	1.60 ***	0.09	1.48 – 1.85	1.60 ***	0.09	1.48 – 1.84
C x R	1.60 ***	0.09	1.49 – 1.82	1.62 ***	0.09	1.51 – 1.85
D x R	0.50 ***	0.03	0.44 – 0.55	0.49 ***	0.03	0.43 – 0.56
Bot x R	0.90 *	0.04	0.81 – 0.98	0.90 *	0.04	0.81 – 0.99
TFT x Bot	0.47 *	0.16	0.26 – 0.89	0.46 *	0.16	0.24 – 0.86
C x Bot	0.42 *	0.15	0.23 – 0.82	0.42 *	0.15	0.22 – 0.84
D x Bot	0.89	0.30	0.47 – 1.70	0.90	0.30	0.45 – 1.70
TFT x Bot x R	0.96	0.07	0.82 – 1.09	0.96	0.07	0.83 – 1.09
C x Bot x R	0.77 ***	0.06	0.66 – 0.87	0.77 ***	0.06	0.65 – 0.87
D x Bot x R	1.44 ***	0.11	1.24 – 1.71	1.44 ***	0.12	1.22 – 1.68
Age (standardized)				0.94	0.06	0.84 – 1.05
Male				0.74 *	0.09	0.57 – 0.94
Observations	21180			20990		
Marginal R^2 / Conditional R^2	0.310 / 0.854			0.315 / 0.856		

* *p<0.05* ** *p<0.01* *** *p<0.001*

Table S9. *Manipulation phase* models discussed in main text, Study 2, related to Figure 3. Cooperation decisions made in the *manipulation phase* (i.e., the first phase) of Study 2. Logistic generalized linear models with random intercepts at the participant level and random slopes for round in the phase. *Round in the phase* ranges from 0 (round 1) to 9 (round 10). Model 2 contains control terms for participant demographics. We computed bootstrapped confidence intervals using the percentile bootstrap method, with N = 1,000 resamples. 19 participants were dropped from this model for missingness on age and/or gender (see Table S1).

	Model 1: Manipulation phase			Model 2: Spillover phase		
Predictors	*HR*	*SE*	*Bootstrapped 95% CI*	*HR*	*SE*	*Bootstrapped 95% CI*
Manipulation phase partner (MPP) played Tit for Tat (TFT)	0.45 ***	0.03	0.39 – 0.52	0.61 ***	0.05	0.43 – 0.66
MPP always cooperated (C)	0.54 ***	0.04	0.47 – 0.62	0.79 *	0.07	0.63 – 0.96
MPP always defected (D)	1.27 ***	0.08	1.16 – 1.39	1.06	0.09	0.83 – 1.26
MPP was a bot (B)	1.57 ***	0.08	1.43 – 1.73	1.39 ***	0.08	1.30 – 1.77
Observations	2118			2118		
R^2 Nagelkerke	0.156			0.681		

* *p*<0.05   ** *p*<0.01   *** *p*<0.001

Table S10. Hazard of defection by condition, Study 2, related to Figure 3. Time to first defection by experimental condition. Cox proportional hazards models. Model 2 includes a frailty term to account for participants belonging to a dyad with a real other participant. The variance ($\sigma^2 = 1.25$) of the frailty term was significant ($p < 0.001$). We computed bootstrapped confidence intervals using the percentile bootstrap method, with N = 1,000 resamples.

Predictors	Model 1: Gender *OR*	*SE*	*Bootstrapped 95% CI*	Model 2: Age *OR*	*SE*	*Bootstrapped 95% CI*
Intercept	1.11	0.20	0.78 – 1.54	1.28	0.22	0.92 – 1.75
Partner played Tit for Tat (TFT)	4.09 ***	1.04	2.45 – 6.56	4.15 ***	1.05	2.56 – 6.64
Partner always cooperated (C)	2.59 ***	0.66	1.61 – 4.26	2.57 ***	0.66	1.58 – 4.18
Partner always defected (D)	0.87	0.21	0.57 – 1.50	0.88	0.21	0.57 – 1.48
Partner was a bot (Bot)	0.69	0.18	0.43 – 1.16	0.52 **	0.12	0.34 – 0.85
Round in the phase (R)	0.80 ***	0.03	0.74 – 0.86	0.79 ***	0.03	0.73 – 0.84
Male (ref: Female)	1.02	0.18	0.72 – 1.39	0.74 *	0.09	0.57 – 0.94
Age (standardized)	0.94	0.06	0.84 – 1.06	0.91	0.08	0.77 – 1.07
Bot x R	0.89 *	0.05	0.79 – 0.99	0.90 *	0.04	0.81 – 0.99
TFT x R	1.60 ***	0.09	1.49 – 1.84	1.60 ***	0.09	1.48 – 1.84
C x R	1.62 ***	0.09	1.50 – 1.85	1.62 ***	0.09	1.50 – 1.85
D x R	0.49 ***	0.03	0.44 – 0.56	0.49 ***	0.03	0.44 – 0.56
TFT x Bot	0.46 *	0.16	0.23 – 0.86	0.45 *	0.16	0.24 – 0.87
C x Bot	0.41 *	0.15	0.21 – 0.81	0.42 *	0.15	0.22 – 0.84
D x Bot	0.90	0.30	0.45 – 1.73	0.89	0.30	0.46 – 1.66
TFT x Bot x R	0.96	0.07	0.83 – 1.09	0.96	0.07	0.83 – 1.09
C x Bot x R	0.77 ***	0.06	0.65 – 0.87	0.77 ***	0.06	0.66 – 0.88
D x Bot x R	1.44 ***	0.12	1.21 – 1.67	1.44 ***	0.12	1.21 – 1.68
Male x R	0.98	0.04	0.91 – 1.05			
Male x Bot	0.54 *	0.13	0.35 – 0.88			
Male x Bot x R	1.03	0.05	0.93 – 1.14			
Age x R				0.99	0.02	0.95 – 1.03
Age x Bot				1.08	0.13	0.86 – 1.37
Age x Bot x R				0.98	0.03	0.93 – 1.03
Observations	20990			20990		
Marginal R^2 / Conditional R^2	0.315 / 0.856			0.315 / 0.856		

* $p<0.05$ ** $p<0.01$ *** $p<0.001$

Table S11. Interactions with gender, age, and being paired with a bot, *Manipulation phase*, Study 2, related to Figure 3. Logistic generalized linear models with random intercepts at the participant level and random slopes for round in the phase. *Round in the phase* ranges from 0 (round 1) to 9 (round 10). We computed bootstrapped confidence intervals using the percentile bootstrap method, with N = 1,000 resamples. 19 participants were dropped from this model for missingness on age and/or gender (see Table S1).

	Model 1			Model 2		
Predictors	*OR*	*SE*	*Bootstrapped 95% CI*	*OR*	*SE*	*Bootstrapped 95% CI*
Intercept	0.62 ***	0.07	0.51 – 0.78	0.44 ***	0.07	0.33 – 0.59
Manipulation phase partner (MPP) played Tit for Tat (TFT)	1.93 ***	0.24	1.51 – 2.38	1.97 ***	0.25	1.56 – 2.45
MPP always cooperated (C)	1.60 ***	0.20	1.26 – 1.98	1.68 ***	0.21	1.32 – 2.12
MPP always defected (D)	1.05	0.13	0.84 – 1.33	1.06	0.13	0.85 – 1.33
MPP was a bot (Bot)	0.74 ***	0.07	0.64 – 0.89	0.77 *	0.08	0.64 – 0.93
Round in the phase (R)	0.76 ***	0.03	0.69 – 0.82	0.75 ***	0.03	0.69 – 0.82
Bot x R	1.05	0.03	0.99 – 1.11	1.05	0.03	0.99 – 1.11
TFT x R	0.89 **	0.04	0.83 – 0.97	0.89 **	0.04	0.82 – 0.97
C x R	0.89 **	0.04	0.82 – 0.97	0.89 *	0.04	0.83 – 0.97
D x R	0.88 **	0.04	0.81 – 0.96	0.88 **	0.04	0.81 – 0.95
Cooperated this round of the manipulation phase	2.96 ***	0.19	2.58 – 3.30	2.95 ***	0.18	2.59 – 3.30
Age (standardized)				0.88 **	0.04	0.82 – 0.95
Male				1.10	0.09	0.95 – 1.30
Suspicion of the manipulation phase partner				1.02	0.03	0.97 – 1.07
Suspicion of the spillover phase partner				1.06 **	0.02	1.02 – 1.10
Observations	21178			20988		
Marginal R^2 / Conditional R^2	0.043 / 0.909			0.045 / 0.910		

** p<0.05   ** p<0.01   *** p<0.001*

Table S12. *Spillover phase* models discussed in main text, Study 2, related to Figure 3. Cooperation decisions made in the *spillover phase* (i.e., the second phase) of Study 2, based on the manipulations that occurred in the *manipulations phase* (i.e., the first phase). Logistic generalized linear models with random intercepts at the dyad and participant level and random slopes for round in the phase. *Round in the phase* ranges from 0 (round 1) to 9 (round 10). Model 2 contains control terms for participant demographics and suspicion of the manipulation phase and spillover phase partner. We computed bootstrapped confidence intervals using the percentile bootstrap method, with N = 1,000 resamples. 19 participants were dropped from this model for missingness on age and/or gender (see Table S1).

Predictors	Model 1: Gender			Model 2: Age		
	OR	*SE*	*Bootstrapped 95% CI*	*OR*	*SE*	*Bootstrapped 95% CI*
Intercept	0.57 ***	0.07	0.45 – 0.72	0.60 ***	0.07	0.48 – 0.75
Manipulation phase partner (MPP) played Tit for Tat (TFT)	1.95 ***	0.24	1.52 – 2.42	1.97 ***	0.24	1.55 – 2.44
MPP always cooperated (C)	1.61 ***	0.20	1.25 – 2.01	1.62 ***	0.20	1.26 – 2.03
MPP always defected (D)	1.02	0.13	0.81 – 1.28	1.02	0.13	0.82 – 1.29
MPP was a bot (Bot)	0.77 *	0.09	0.62 – 0.95	0.75 **	0.07	0.65 – 0.89
Round in the phase (R)	0.78 ***	0.04	0.71 – 0.85	0.75 ***	0.03	0.69 – 0.82
Cooperated this round of the manipulation phase	2.96 ***	0.19	2.58 – 3.34	2.96 ***	0.19	2.60 – 3.32
Male (ref: Female)	1.23	0.16	0.96 – 1.55	1.10	0.09	0.94 – 1.29
Age (standardized)	0.87 ***	0.04	0.81 – 0.94	0.91	0.06	0.80 – 1.03
Bot x R	1.04	0.04	0.97 – 1.12	1.05	0.03	0.99 – 1.11
TFT x R	0.89 **	0.04	0.83 – 0.97	0.89 **	0.04	0.82 – 0.97
C x R	0.89 **	0.04	0.83 – 0.97	0.90 *	0.04	0.83 – 0.98
D x R	0.88 **	0.04	0.81 – 0.95	0.88 **	0.04	0.81 – 0.95
Male x Bot	0.95	0.17	0.70 – 1.31			
Male x R	0.93	0.04	0.86 – 1.01			
Male x Bot x R	1.02	0.06	0.91 – 1.15			
Age x Bot				0.89	0.08	0.76 – 1.06
Age x R				1.02	0.02	0.98 – 1.07
Age x Bot x R				0.98	0.03	0.92 – 1.03
Observations	20988			20988		
Marginal R^2 / Conditional R^2	0.044 / 0.911			0.044 / 0.910		

* *p<0.05* ** *p<0.01* *** *p<0.001*

Table S13. Interactions with gender, age, and being paired with a bot, *Spillover phase,* Study 2, related to Figure 3. Logistic generalized linear models with random intercepts at the dyad and participant level and random slopes for round in the phase. *Round in the phase* ranges from 0 (round 1) to 9 (round 10). We computed bootstrapped confidence intervals using the percentile bootstrap method, with N = 1,000 resamples. 19 participants were dropped from this model for missingness on age and/or gender (see Table S1).

Predictors	Model 1: Ratings of the *manipulation phase partner* (a bot or ostensible human)			Model 2: Ratings of the *spillover phase partner* (a real other participant		
	Est.	*SE*	*Bootstrapped 95% CI*	*Est.*	*SE*	*Bootstrapped 95% CI*
Intercept	3.72 ***	0.08	3.55 – 3.90	4.02 ***	0.10	3.84 – 4.22
Manipulation phase partner (MPP) was a bot	-2.03 ***	0.07	-2.18 – -1.89	0.11	0.09	-0.07 – 0.28
MPP played Tit for Tat	0.19	0.10	-0.01 – 0.40	-0.22	0.13	-0.46 – 0.02
MPP always defected	-0.16	0.10	-0.38 – 0.04	-0.65 ***	0.13	-0.89 – -0.40
MPP always cooperated	-0.33 **	0.10	-0.52 – -0.11	-0.60 ***	0.13	-0.85 – -0.37
Observations	2118			2118		
R^2 / R^2 adjusted	0.273 / 0.272			0.017 / 0.015		

Table S14. Rating of the partner as "a real person", for both the manipulation phase and spillover phase partner, Study 2, related to STAR Methods. Linear regressions of participants' suspicion (lower values indicate greater suspicion/less rating the partner as a real person) of their manipulation and spillover phase partners. We computed bootstrapped confidence intervals using the percentile bootstrap method, with N = 1,000 resamples.

Predictors	Model 1: Ratings of the *manipulation phase partner* (a bot or ostensible human) Est.	SE	Bootstrapped 95% CI	Model 2: Ratings of the *spillover phase partner* (a real other participant) Est.	SE	Bootstrapped 95% CI
Intercept	3.71 ***	0.10	3.46 – 3.92	3.91 ***	0.13	3.68 – 4.15
Manipulation phase partner (MPP) was a bot (Bot)	-2.00 ***	0.15	-2.27 – -1.71	0.35	0.18	0.00 – 0.69
MPP played Tit for Tat (TFT)	0.38 **	0.15	0.06 – 0.73	-0.06	0.18	-0.38 – 0.28
MPP always defected (D)	-0.27	0.15	-0.61 – 0.12	-0.38 *	0.18	-0.73 – -0.03
MPP always cooperated (C)	-0.37 *	0.15	-0.71 – -0.03	-0.56 **	0.18	-0.91 – -0.20
Bot x TFT	-0.36	0.21	-0.75 – 0.02	-0.32	0.25	-0.80 – 0.16
Bot x D	0.19	0.21	-0.27 – 0.58	-0.52 *	0.26	-1.03 – -0.05
Bot x C	0.09	0.21	-0.33 – 0.49	-0.09	0.25	-0.62 – 0.44
Observations	2118			2118		
R^2 / R^2 adjusted	0.276 / 0.273			0.017 / 0.015		

Table S15. Rating of the partner as "a real person", for both the manipulation phase and spillover phase partner, with interactions between conditions, Study 2, related to STAR Methods. Linear regressions of participants' suspicion (lower values indicate greater suspicion/less rating the partner as a real person) of their manipulation and spillover phase partners. We computed bootstrapped confidence intervals using the percentile bootstrap method, with N = 1,000 resamples. See Table S14 for models with just the main effects.

Predictors	Model 1: Empathy toward the MPP Est.	SE	Bootstrapped 95% CI	Model 2: Key model in the spillover phase, *without* total empathy toward the MPP OR	SE	Bootstrapped 95% CI	Model 3: Key model in the spillover phase, *with* total empathy toward the MPP OR	SE	Bootstrapped 95% CI
Intercept	3.38 ***	0.06	3.28 – 3.49	0.62 ***	0.07	0.50 – 0.76	0.42 ***	0.06	0.31 – 0.56
Manipulation phase partner (MPP) played Tit for Tat (TFT)	0.06	0.08	-0.09 – 0.21	1.93 ***	0.24	1.53 – 2.39	1.94 ***	0.24	1.53 – 2.43
MPP always cooperated (C)	0.03	0.08	-0.12 – 0.18	1.60 ***	0.20	1.26 – 1.97	1.59 ***	0.20	1.27 – 2.01
MPP always defected (D)	-0.07	0.08	-0.22 – 0.08	1.05	0.13	0.84 – 1.33	1.05	0.13	0.85 – 1.35
MPP was a bot (B)	-0.91 ***	0.06	-1.02 – -0.80	0.74 ***	0.07	0.65 – 0.89	0.82 *	0.08	0.72 – 1.00
Round in the phase (R)				0.76 ***	0.03	0.70 – 0.82	0.75 ***	0.03	0.70 – 0.82
Cooperated this round of the manipulation phase				2.96 ***	0.19	2.58 – 3.33	2.90 ***	0.18	2.51 – 3.23
B x R				1.05	0.03	0.98 – 1.11	1.05	0.03	0.99 – 1.11
TFT x R				0.89 **	0.04	0.83 – 0.97	0.89 **	0.04	0.83 – 0.97
C x R				0.89 **	0.04	0.83 – 0.97	0.89 **	0.04	0.83 – 0.96
D x R				0.88 **	0.04	0.81 – 0.95	0.88 **	0.04	0.81 – 0.95
Empathy toward the MPP							1.12 ***	0.04	1.06 – 1.19
Observations	2118			21178			21178		
R^2 / R^2 adjusted	0.115 / 0.114			0.043 / 0.909			0.044 / 0.909		
AIC	7023.238			13839.718			13827.656		

* *p<0.05* ** *p<0.01* *** *p<0.001*

Table S16. Mediation analysis: the effect of being paired with a bot in the manipulation phase on empathic concern, perspective taking, and cooperation rate in the spillover phase in Study 2, related to STAR Methods. Model 1 is a linear regression showing that being in the bot condition significantly predicted lower total empathy toward the (bot) manipulation phase partner (the mediator). Model 2 shows our key model for the spillover phase before total empathy toward the manipulation phase partner is added to the model. Model 3 is the key model but with total empathy toward the manipulation phase partner added. Empathy toward the MPP predicts cooperation in the spillover phase and partially mediates the effect of bot (OR = 0.74 [0.65, 0.89], $p < 0.001$ in Model 2 to OR = 0.82 [0.72, 1.00], $p < 0.05$ in Model 3). We computed bootstrapped confidence intervals using the percentile bootstrap method, with N = 1,000 resamples.
